# Associations between dietary patterns and cardiovascular, kidney and metabolic risk markers in the Cooperative Health Research in South Tyrol study

**DOI:** 10.1007/s00394-026-03992-y

**Published:** 2026-05-29

**Authors:** Essi Hantikainen, Sophie Berger, Giulia Barbieri, Peter P. Pramstaller, Vanessa Garcia-Larsen, Martin Gögele, Cristian Pattaro, Rebecca Lundin, Francisco S. Domingues

**Affiliations:** 1 Institute for Biomedicine, Eurac Research, Bolzano, Italy; 2 Department of Nutritional Sciences, University of Vienna, Vienna, Austria; 3 Program in Human Nutrition, Department of International Health, Bloomberg School of Public Health at Johns Hopkins University, Baltimore, MD USA

**Keywords:** Diet, Cardiovascular-renal-metabolic health, Noncommunicable disease, Public health

## Abstract

**Purpose:**

Cardiovascular-kidney metabolic syndrome (CKM) represents an increasingly prevalent public health concern. Optimal strategies to prevent CKM-related outcomes are greatly needed. We assessed the association between various dietary patterns and CKM biomarkers in a general population study.

**Methods:**

We used cross-sectional data from 8066 adult participants of the Cooperative Health Research in South Tyrol (CHRIS) study. Self-reported dietary intake was assessed through the semi-quantitative GA^2^LEN Food Frequency questionnaire. We derived six established dietary pattern indices, and population-specific dietary patterns through principal component analysis. CKM biomarkers included: systolic and diastolic blood pressure, glycated haemoglobin, visceral fat, HDL, LDL and total cholesterol, triglycerides, creatinine-based estimated glomerular filtration rate, and urinary albumin-to-creatinine ratio. We investigated associations between each dietary index and CKM risk marker using multivariable linear regression models. We further examined potential effect modification by sex, body mass index, physical activity and smoking habit.

**Results:**

Higher adherence to healthy dietary patterns rich in fruits, vegetables, whole grains, and low in saturated fats was associated with more favorable CKM marker levels. E.g, when comparing the fourth to the first quartile the healthy plant-based dietary index was associated with lower total and LDL cholesterol (β ≈ −  5.0 mg/dL, *p* <  0.001), lower blood pressure (β= − 1.24 mmHg, *p* = 0.001) and higher eGFR (β = 1.24 ml/min/1.73m^2^, *p* = 0.001). In contrast, the unhealthy plant based and a Western style diet were associated with higher SBP (β  =  1.23–1.89 mmHg, *p* < 0.01), amongst others. For some markers, associations tended to be stronger in men, individuals with overweight or obesity, those with lower physical activity, and smokers (P_interaction_< 0.05).

**Conclusions:**

Adhering to a healthy plant rich diet low in saturated fats, added sugars and processed foods was associated with more favorable levels of CKM biomarkers, including blood lipids, blood pressure, glycated hemoglobin and eGFR.

**Supplementary Information:**

The online version contains supplementary material available at 10.1007/s00394-026-03992-y.

## Introduction

Non-communicable diseases such as type 2 diabetes mellitus (T2DM), cardiovascular disease (CVD), and chronic kidney disease (CKD) are increasingly prevalent in many parts of the world [1], representing a major public health concern. Epidemiological studies consistently show an interdependence between cardiovascular, renal, and metabolic diseases [2]. This led the American Heart Association (AHA) to introduce the concept of a combined disease condition known as Cardiovascular-Kidney and Metabolic Syndrome (CKM) [3].

The term encompasses the progressive stages of risk factors, such as obesity, diabetes, CKD and CVD, where various biological and lifestyle exposures contribute to the accumulation of excess and dysfunctional adipose tissue, resulting in inflammation, oxidative stress and insulin resistance. Their presence can lead to hypertension, hypertriglyceridemia, metabolic syndrome, T2DM and CKD, as well as subclinical or clinical CVD, all of which increase risk of premature mortality and other co-morbidities [3, 4]. Given its high prevalence, it is critical to identify modifiable lifestyle CKM-risk factors at the general population level [3].

Adherence to healthy dietary patterns rich in plant-based foods, such as the Mediterranean diet or the Alternative Healthy Eating Index (AHEI), have been associated with CKM risk markers, such as improved lipid profile [5], lower blood pressure [6], lower insulin sensitivity [7], and lower risk of cardiometabolic and renal diseases [8, 9]. In contrast, a Western-style diet, characterized by high consumption of animal-based and processed foods, has been unfavorably linked to cardiometabolic traits, such as higher waist circumference, systolic and diastolic blood pressure, fasting glucose and insulin, as well as higher incidence of kidney disease [10–12]. Recently, the consumption of ultra-processed foods has gained great attention: their high content of sugar, fat and salt contributes to hyper-palatability and energy density. These characteristics may influence eating behavior by promoting overconsumption and reducing satiety. Moreover, the structural and compositional modifications that appear due to intensive chemical and physical processing might further enhance the rate of digestion and nutrient absorption, potentially influencing postprandial metabolism and satiety [13, 14]. However, studies have reported mixed findings regarding ultra-processed foods impact on cardiometabolic traits and renal health [15–18]. Since diet is a modifiable lifestyle factor, dietary interventions might be particularly effective in the early prevention of hypertension, hypertriglyceridemia, impaired glycemia, all of which are common CKM risk markers [19].

Although several epidemiological and clinical studies have explored the relationship between various diets and the risk of cardiometabolic and renal disease, the interconnected nature of these conditions and the growing attention towards CKM underscores the importance of evaluating cardiometabolic and renal endpoints together. Assessing disease risk biomarkers as intermediate endpoints allows for deeper investigation into disease development, which may support the development of prevention strategies targeting subclinical stages of CKM. Our investigation aimed to assess the associations of various dietary patterns on a range of biomarkers associated with CKM risk in the population-based Cooperative Health and Research in South Tyrol (CHRIS) study.

## Methods

### Study population

The CHRIS study is a population-based cohort study that was established to study genetic and lifestyle determinants of health and healthy aging in an Alpine, single administrative district of the Val Venosta/Vinschgau, in South Tyrol, Italy [20]. The cohort comprises 13,393 adults aged 18 and over recruited from 13 municipalities. Baseline visits were conducted from 2011 to 2018, collecting socio-demographic, health, lifestyle, and exposure data from questionnaires, interviews, and instrumental examinations [20, 21]. The study was approved by the Ethics Committee of the Healthcare System of the Autonomous Province of Bolzano/Bozen - South Tyrol.

### Outcome assessment

Anthropometric and blood pressure measurements as well as blood and urine for standard laboratory tests and biobanking were collected at the study center following overnight fasting [20]. Blood and urine samples were collected at study center, and pre-analytical sample processing was performed immediately at study center as previously described [21, 22]. Samples were then shipped to the hospital laboratory at room temperature or at 4 °C, as appropriate, to be analyzed on the same day. Sample temperature was monitored and recorded during transport by electronic thermometers. Outcomes reflecting key aspects of CKM health included: systolic (SBP) and diastolic (DBP) blood pressure, glycated haemoglobin (HbA1c), visceral fat, lipid profile (total, HDL, LDL cholesterol, triglycerides), estimated glomerular filtration rate (eGFR), and urinary albumin to creatinine ratio (UACR). Visceral fat was measured with the OMRON BF508 bioelectrical impedance analysis device, reporting the Visceral Fat Level index (range 1–30). Quantile normalization to the last laboratory assay was used to account for changes in measurement methods [23]. eGFR was estimated from serum creatinine using the 2021 CKD-EPI equation [24], implemented via the ‘nephro’ R package, *v1.3* [25].

### Dietary assessment

Dietary intake was assessed using the self-administered, semi-quantitative Global Allergy and Asthma European Network (GA^2^LEN) food frequency questionnaire (FFQ), which was designed as a single, standardized, internationally validated instrument to ascertain dietary intake, facilitating international comparisons [26]. Dietary intake assessment has been described in detail elsewhere [27]. The FFQ records the consumption of 229 foods and beverages categorized into 32 sections. Participants were asked to report their average consumption frequencies over the past 12 months for each item. Food portion sizes were estimated using the Food Standard Agency’s Food Portion Sizes Guidelines [28]. Consumption frequency of specific food items was transformed into grams per day, from which total energy intake (TEI) and macro- and micronutrients were estimated using the McCance & Widdowson’s Food Composition Table [29].

The following established dietary quality indices were derived and included in the analyses for their role in CKM-associated metabolic conditions: the AHEI-2010 [30]; the Mediterranean diet index (MED) [31]; and the Dietary Approach to Stop Hypertension index (DASH) [32]. We estimated three versions of a plant-based diet index [33]: an overall plant-based diet index (PDI) (higher scoring of any kind of plant based food); a healthy plant-based diet index (hPDI) (higher scoring of whole grains, fruits, vegetables, nuts, legumes, vegetable oils, tea/coffee); and an unhealthy plant-based diet index (uPDI) (higher scoring of refined grains, sweets, sugary beverages, potatoes). Moreover, we estimated Ultra-Processed Food Intake (UPF) using the NOVA scoring system [13]. The UPF group was identified as the food items in group four of the NOVA system and expressed in %-energy per day. Through principal component analysis (PCA) with orthogonal varimax rotation [34] we derived dietary patterns specific to the study population: we selected the first two rotated principal components (RCs), which we labelled as a “Western style dietary pattern” (RC1) and a “Prudent style dietary pattern” (RC2), and which explained 12% and 10% of the total variance, respectively. The factor loadings for the first two components are presented in Supplementary Fig. 1. The details of the dietary indices and the PCA are described in Supplementary Text S1 and Supplementary Tables S1 and S2.

### Confounder assessment

The following potential confounders were selected based on evidence linking them to the dietary indices and outcomes under study: age (years); sex; educational level (primary school or no title; lower secondary school; vocational school; upper secondary school; and university or higher); smoking habit (never, former, current smoker) [35]; physical activity (low; moderate; high activity) assessed using the short version of the International Physical Activity Questionnaire [36] and the metabolic equivalent of task expressed in total MET-minutes/week [37]; body mass index (BMI, kg/m^2^); and following a special diet for reasons of managing health conditions or weight (yes; no), as reported through the FFQ. Moreover, awareness of comorbidities including hypertension, diabetes and dyslipidemia was assessed by combining self-reported information collected through interviews with medication information collected by scanning barcodes of boxes of medications used within the seven days prior to the study center visit. Awareness of kidney disease was assessed using a self-reported kidney disease questionnaire [38], asking whether the participants had ever received a diagnosis of kidney diseases, including reduced kidney function or renal failure.

### Exclusion criteria

We included data from all 8,829 participants to whom the GA^2^LEN FFQ was administered from May 5th, 2014, until the end of 2018. To reduce missing information and minimize data entry errors, data acquisition was automatized by scanning paper questionnaires and trained personnel assisted the participants at their study visit [27]. We excluded from the analysis 120 individuals with > 20% missing FFQ items or who were classified in the < 0.5th or > 99.5th percentile of the TEI/basal metabolic rate ratio [39], with the latter enabling us to further rationalize the extreme intake in relation to basic physiological functions. Remaining missing values in the FFQ have previously not shown any consistent patterns and 99% of the participants had < 5% missing items [27]. All remaining missing values in the FFQ were therefore treated as zero intake. Finally, we excluded 643 non-fasting individuals, leaving 8066 participants for the statistical analyses.

### Statistical analysis

Prior to the analyses, all dietary indices, except UPF (as this was already expressed in %-energy), were adjusted for TEI (kcal) using the nutrient residual model [40]. HbA1c, triglycerides and UACR were transformed to the natural logarithm to address skewness. Dietary scores were analyzed as continuous variables (per 1-SD, increase, after applying z-standardization), as well as after categorizing the exposures into sex-specific quartiles. We fitted multivariable adjusted linear regression models to investigate associations between the various dietary scores (exposure variables) and CKM markers (outcome variables) and estimated regression coefficients (β) with 95% Confidence Intervals (95% CI). All analyses were adjusted for age, sex, physical activity, education, smoking, special diet, BMI, medication or self-report for hypertension, diabetes, and dyslipidemia, self-reported kidney disease, and TEI. All models were adjusted for alcohol intake (g/day), except for AHEI, MED, RC1, and RC2, which already include alcohol intake in their calculation. UPF index was additionally adjusted for AHEI to account for overall dietary habits that are not accounted for directly in the UPF as it only reflects the industrial processing of foods [41]. To explore similarities in the associations between dietary scores and CKM markers, we additionally compared standardized effect sizes across all models and applied hierarchical clustering using the Euclidean distance to group dietary scores with similar association patterns using the ‘pheatmap’ R package, v1.0.12. More specifically, for each dietary score *i* the standardized effect sizes in the respective model for CKM marker *j* are represented in vector *s*_ij_ = (*s*_i1_,…, ,*s*_im_), where *m* is the total number of CKM markers. For any two dietary scores a, b, the Euclidean distance between the respective vectors *s*_aj_, *s*_bj_ is $\:d({s}_{aj},{s}_{bj})=\sqrt{{({s}_{a1}-{s}_{b1})}^{2}+\dots\:+{({s}_{am}-{s}_{bm})}^{2}}$. All* p*-values were adjusted for multiple comparisons at the model level using the Benjamini Hochberg false discovery rate (FDR) method and adjusted* P*-values < 0.05 were considered statistically significant.

Linear trends were further examined by including the quartile variable as continuous variables in the models.* P*-values from the trend evaluation were not corrected for multiple comparisons because the test for linear trend represents a single, prespecified hypothesis used as a confirmatory assessment of the dose–response shape and therefore unadjusted* P*-values < 0.05 were considered to be statistically significant.

We also investigated potential effect modification on the multiplicative scale. To do so we fitted an interaction term between the dietary variables and the potential dichotomized effect modifiers, which were sex, overweight/obesity (yes vs. no), smoking (never/former vs. current), and physical activity (low vs. high, based on the median reported activity expressed in MET h/day). We additionally conducted stratified analyses to further explore the association of diet and CKM markers within the specified subgroups. For the interaction analysis we considered unadjusted* P*-values < 0.05 to be statistically significant.

All analyses were performed using the R statistical software v4.4.2.

## Results

Participants’ characteristics are presented in Table 1. The mean age was 45.4 years (SD = 16.8, ranging from 18 to 94 years, Supplementary Fig.S2 ) and 54.5% of them were female. An education level of high school or higher was reported by 32.6% of the participants, 17.5% reported to be currently smoking, and 50% were classified as being highly physically active. Based on self-report or medication information, 23.3%, 21.9%, 8.6%, and 2.6% of study participants reported hypertension, dyslipidemia, kidney disease, and T2DM, respectively.

**Table 1:** Study participants’ characteristics

Characteristic	Overall	Males	Females
Sample size	*n* = 8066	*n* = 3673	*n* = 4393
*Sex, n (%)*			
Male	3673 (45.5)		
Female	4393 (54.5)		
Age, years, mean (SD)	45.4 (16.8)	45.9 (16.7)	44.9 (16.8)
BMI, kg/m^2^, mean (SD)	25.9 (4.6)	26.4 (4.0)	25.4 (5.0)
Total energy intake, kcal/day, mean (SD)	1960 (613)	2030 (655)	1890 (568)
Alcohol, g/day, mean (SD)	6.20 (8.2)	9.35 (9.87)	3.57 (5.21)
Special diet, yes, n (%)	573 (7.1)	215 (5.9)	358 (8.1)
*Physical activity, IPAQ score, n (%)*			
Low^*a*^	1274 (15.8)	579 (15.8)	695 (15.8)
Moderate^*a*^	2145 (26.6)	805 (21.9)	1340 (30.5)
High^*a*^	4036 (50.0)	1979 (53.9)	2057 (46.8)
Missing information	611 (7.6)	310 (8.4)	301 (6.9)
*Education, n (%)*			
Primary school	800 (9.9)	284 (7.7)	516 (11.7)
Lower secondary school	1315 (16.3)	509 (13.9)	806 (18.3)
Vocational school	3306 (41.0)	1928 (52.5)	1378 (31.4)
Upper secondary school	1874 (23.2)	681 (18.5)	1193 (27.2)
University or higher	755 (9.4)	262 (7.1)	493 (11.2)
Missing information	16 (0.2)	9 (0.2)	7 (0.2)
*Smoking, n (%)*			
Never smoker	4440 (55.0)	1842 (50.1)	2598 (59.1)
Former smoker	2177 (27.0)	1138 (31.0)	1039 (23.7)
Current smoker	1415 (17.5)	678 (18.5)	737 (16.8)
Missing information	34 (0.4)	15 (0.4)	19 (0.4)
*Comorbidity, yes, n (%)*			
Hypertension^b^	1881 (23.3)	906 (24.7)	975 (22.2)
Dyslipidemia^b^	1764 (21.9)	830 (22.6)	934 (21.3)
Diabetes^b^	212 (2.6)	95 (2.6)	117 (2.7)
Kidney disease^c^	695 (8.6)	248 (6.8)	447 (10.2)

SD, standard deviation; BMI, body mass index; IPAQ, International physical activity questionnaire

^*a*^Physical activity was classified as high in case of (a) vigorous-intensity activity on at least 3 days and accumulating at least 1500 metabolic equivalent of task (MET)-minutes/week, or (b) 7 or more days of any combination of walking, moderate- or vigorous-intensity activities accumulating at least 3000 MET-minutes/week; as moderate in case of (a) 3 or more days of vigorous activity of at least 20 min per day, (b) 5 or more days of moderate-intensity activity and/or walking of at least 30 min per day, or (c) 5 or more days of any combination of walking, moderate-intensity or vigorous-intensity activities achieving a minimum of at least 600 MET-minutes/week; and as low in any other case

^b^Comorbidity was defined as self-reported presence of the condition or taking medication to treat the condition

^c^Kidney disease was defined as self-reported presence of the condition

Participants’ characteristics by sex-specific quartiles for each dietary pattern are reported in Supplementary Table S3. In general, women had higher mean values of all healthy dietary patterns, while mean values for uPDI, RC1 and UPF were higher in males. Participants in the highest quartiles of healthy dietary patterns tended to be older, more physically active, and consumed more alcohol as compared to participants in the lowest quartiles. They had a tendency to have a higher educational level, to be non-smokers and to follow a special diet. Moreover, they reported to a higher degree to having been diagnosed with or taking medication for hypertension, lipid disturbances, diabetes mellitus, and chronic kidney disease.

The Spearman correlations between all dietary patterns are presented in Supplementary Fig. S3. Healthy dietary patterns showed positive weak to strong correlations ranging from 0.29 to 0.79, with strongest correlations observed between the Prudent style dietary pattern (RC2) and DASH (*r* = 0.79). UPF showed weak positive correlations with the unhealthy Western style dietary pattern (RC1) (*r* = 0.26) and uPDI (*r* = 0.36) and was negatively correlated with all healthy dietary patterns, with correlation ranging from − 0.07 to -0.46, indicating a poorer dietary profile with increasing UPF intake.

**Fig. 1:**
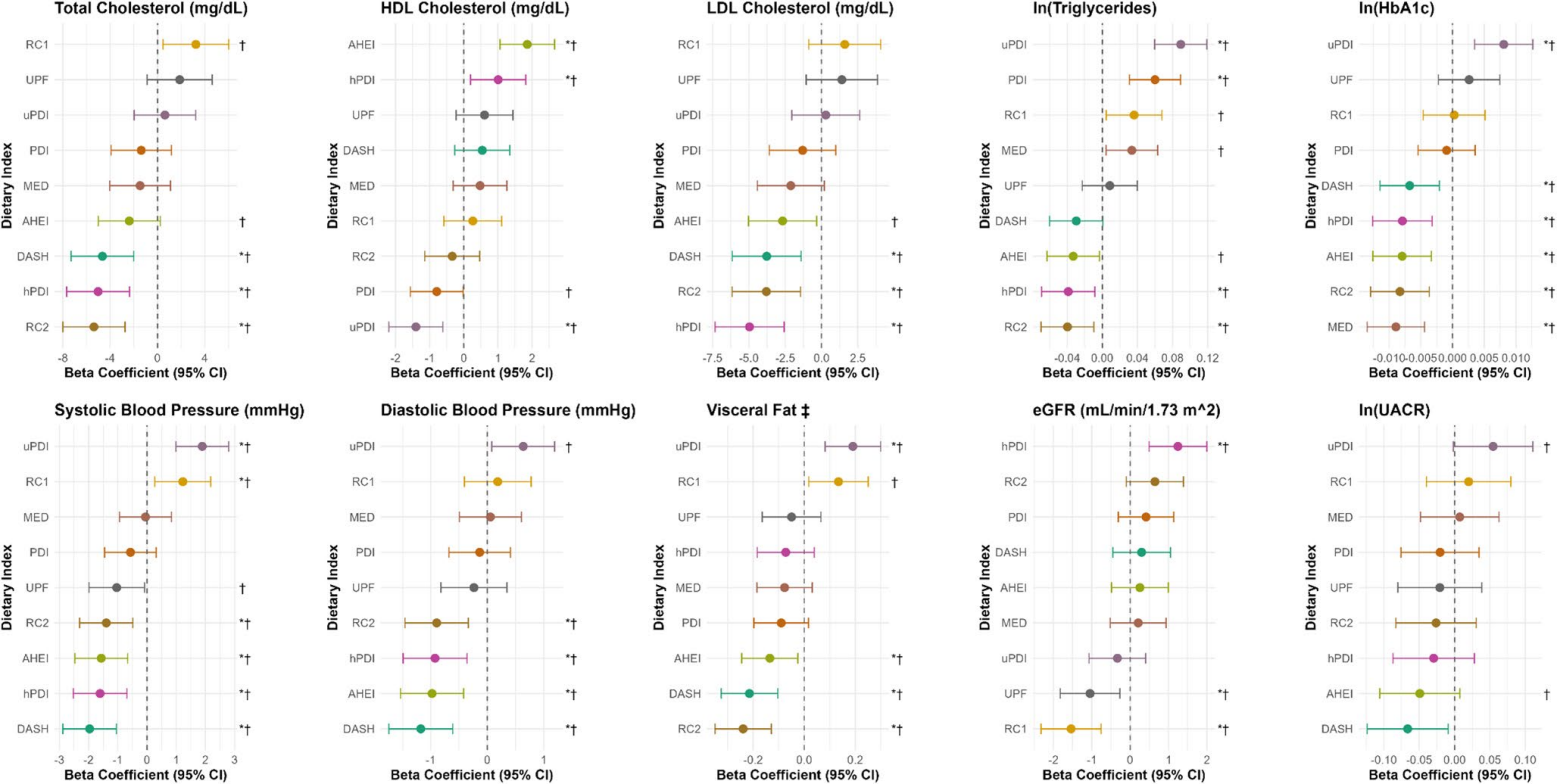
Associations between dietary patterns and cardiovascular-kidney-metabolic risk markers. The forest plots show the regression coefficients comparing quartile four to quartile one for each of the dietary patterns (visually represented by the centers of the error bars), the 95% CIs (visually represented by the error bars). Linear regression models were adjusted for: age, sex, physical activity, education, smoking, special diet, BMI, medication or self-report of hypertension, diabetes mellitus, and dyslipidemia, and self-reported kidney disease, total energy intake and alcohol intake for DASH, PDI, uPDI and hPDI. UPF was additionally adjusted for the AHEI to account for overall dietary quality. *: Two-sided* P*-value < 0.05 after adjustment for multiple comparison using FDR. †: Two-sided P_trend_ < 0.05 (unadjusted for multiple comparison). ‡ Visceral Fat Level index (range 1–30)

**Fig. 2:**
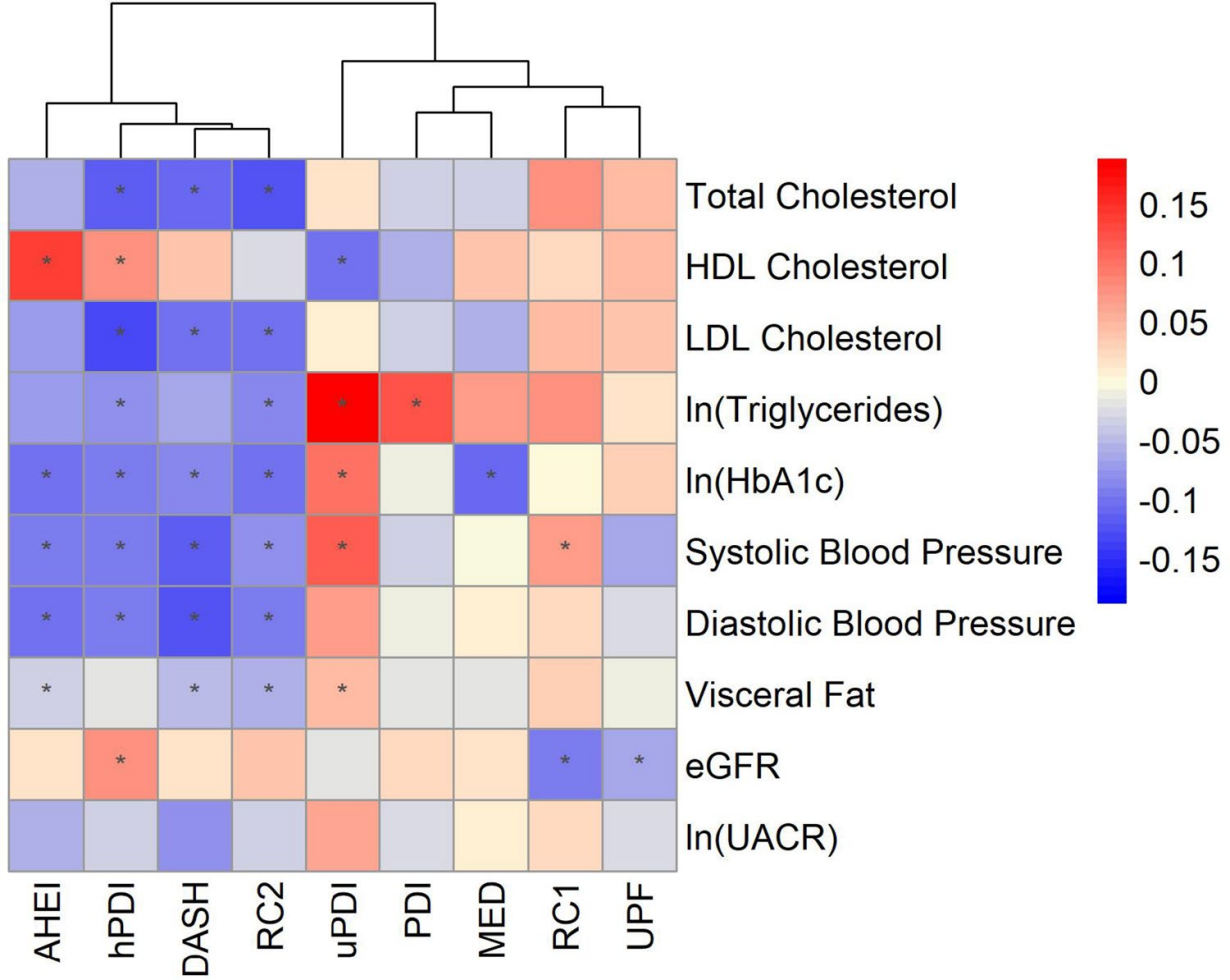
Heatmap presenting effect sizes for the associations between dietary patterns and cardiovascular-kidney-metabolic (CKM) risk markers. Associations were obtained from linear regression models after scaling the CKM risk markers using z-standardisation with the regression coefficients comparing quartile four to quartile one for each of the dietary patterns. Linear regression models were adjusted for: age and sex, physical activity, education, smoking, special diet, BMI, medication or self-report of hypertension, diabetes mellitus, or dyslipidemia, and self-reported kidney disease, total energy intake and alcohol intake for DASH, PDI, uPDI and hPDI. UPF was additionally adjusted for the AHEI to account for overall dietary quality. The asterisk (*) marks significant associations after correction for multiple comparison using FDR. Hierarchical clustering using the Euclidean distance as the similarity measure was applied by clustering rows and columns based on the coefficients describing the associations between dietary patterns with each corresponding CKM marker

When investigating associations with each CKM marker, higher adherence to the healthy dietary patterns (AHEI, DASH, RC2, MED, PDI, hPDI) was associated with more favorable levels of CKM markers, whereas the unhealthy patterns (i.e. uPDI, RC1, UPF) were associated with unfavorable levels of these markers (Figs. 1 and 2, Supplementary Table 4). Multivariable adjusted regression coefficients (β) comparing the fourth to the first quartile with 95% Confidence Intervals (CIs) are presented in Fig. 1 and Supplementary Table 4. When referring to statistically significant results, this implies significance at 0.05 FDR.

For total cholesterol, significant negative associations were observed with β-coefficients for DASH, hPDI and RC2 ranging from − 4.65 mg/dL (95% CI − 7.30, − 2.01) to − 5.37 mg/dL (95% CI − 7.99, − 2.74). For HDL cholesterol, significant positive associations were observed with AHEI (β: 1.86 mg/dL, 95% CI 1.06, 2.66) and HPDI (β: 1.01 mg/dL, 95% CI 0.20, 1.81), and significant negative associations with uPDI (β: − 1.40 mg/dL, 95% CI − 2.19, − 0.61). For LDL cholesterol, significant negative associations were observed for DASH, RC2 and hPDI, which ranged from − 3.77 mg/dL (95% CI − 6.14, − 1.40) to − 4.96 mg/dL (95% CI − 7.33, − 2.58). For triglycerides (log-scale), significant positive associations were observed with PDI (β: 0.06, 95% CI 0.03, 0.09) and uPDI (β: 0.09, 95% CI 0.06, 0.12). Significant negative associations were observed with hDPI (β: − 0.04, 95% CI − 0.07, − 0.01) and RC2 (β: − 0.04, 95% CI − 0.07, − 0.01). For glycated hemoglobin (log-scale) we observed a significant positive association with uPDI (β: 0.01, 95% CI 0.00, 0.01), and negative associations for DASH, hPDI, AHEI, RC2 and MED, with similar effect sizes (β: − 0.01, 95% CI − 0.01, 0.00). For SBP and DBP we observed significant negative associations with RC2, AHEI, hPDI and DASH, with DASH showing the strongest association with both SBP (β: − 1.97 mmHg, 95% CI − 2.88, − 1.05) and DPB (β: − 1.18 mmHg, 95% CI − 1.74, − 0.61). Moreover, uPDI (β: 1.89 mmHg, 95% CI 0.99, 2.80) and RC1 (β: 1.23 mmHg, 95% CI 0.27, 2.19) were significantly positively associated with SBP. For visceral fat, significant positive associations were observed with uPDI (β: 0.19, 95% CI 0.08, 0.30), whereas negative associations were found with AHEI, DASH and RC2, with strongest associations observed for RC2 (β: − 0.24, 95% CI − 0.35, − 0.13). For eGFR, we observed a significant positive association with hPDI (β: 1.24 ml/min/1.73m^2^, 95% CI 0.49, 2.00) and a significant negative association with RC1 (β: − 1.54 ml/min/1.73m^2^, 95% CI − 2.32, − 0.76) and UPF (β: − 1.05 ml/min/1.73m^2^, 95% CI − 1.82, − 0.27). No significant associations were observed with logUACR.

When comparing effect sizes of the associations (Fig. 2, Supplementary Table S5), values were small, with positive associations ranging from 0.01 to 0.19, and negative associations from − 0.01 to − 0.13. We observed the strongest positive associations between unhealthy dietary patterns and triglycerides and strongest negative associations between healthy dietary patterns and LDL as well as total cholesterol. Based on hierarchical clustering of the coefficients representing associations between dietary patterns and CKM markers, associations related to healthy patterns (RC2, AHEI, DASH, hPDI) formed a distinct cluster. We also observed a cluster of associations involving the PDI and MED, RC1 and UPF. The associations with uPDI were slightly distinct from the other dietary patterns, being associated with unhealthier CKM marker levels across various markers. It is noticeable that hPDI is the dietary pattern most associated with favorable CKM marker levels (eight out of the ten risk markers), while uPDI is most associated with unfavorable marker levels (five out of 10 markers). Associations between dietary pattern scores modeled as continuous variables (per 1SD- increment) and the CKM markers confirmed most of these findings (Supplementary Fig. S4, Table S5). However, additional significant associations were observed between RC1, total cholesterol, triglycerides and visceral fat, as well as between MED, LDL and triglycerides.

### Interactions

Associations between all dietary patterns and CKM markers varied strongly by sex (Supplementary Fig. S5) for total cholesterol, LDL cholesterol, triglycerides, glycated hemoglobin, SBP, visceral fat and eGFR, with associations being stronger in males (P_interaction_: < 0.001 to 0.39).

We additionally observed statistically significant interactions between diet and BMI (Supplementary Fig. S6). Among individuals with overweight and obesity, stronger associations were seen between various healthy dietary patterns and total cholesterol, LDL cholesterol, triglycerides, visceral fat (P_interaction_: < 0.001 to 0.031). The association between PDI and eGFR was stronger in normal weight individuals (P_interaction_: 0.020). Differences between normal weight vs. overweight or obesity were further observed between uPDI, RC1 and UPF for total cholesterol (P_interaction_: < 0.001 to 0.005), LDL (P_interaction_: 0.008 to 0.030), triglycerides (P_interaction_ for RC1: 0.031), visceral fat (P_interaction_ for RC1 and uPDI: < 0.001 to 0.035), and logUACR (P_interaction_ for UPF: 0.017).

Interactions between physical activity and various dietary patterns (Supplementary Fig. S7) were observed for HDL cholesterol, visceral fat and logUACR (P_interaction_: < 0.001 to 0.30). The associations were stronger in participants with a physical activity level below the median for RC2, PDI with HDL cholesterol, whereas they were stronger for the high physical activity group for DASH and uPDI. With regards to visceral fat, associations were stronger in individuals with higher physical activity (AHEI, MED).

Only few interactions between dietary patterns and smoking habit were observed (Supplementary Fig. S8). While higher adherence to DASH and RC2 were associated with lower SBP in both subgroups, the association was stronger in smokers (P_interaction_: 0.002 to 0.023). The opposite was observed for RC1, where higher adherence was related to higher SBP in non-smokers, while no association was observed among smokers (P_interaction_: 0.037). RC1 had additionally a stronger positive association with visceral fat in non-smokers (P_interaction_: 0.030). Smoking status further modified the association between UPF and HDL (P_interaction_: 0.003).

## Discussion

We investigated associations of various dietary patterns with several CKM risk markers. We found that higher adherence to unhealthy dietary patterns, specifically the uPDI and the Western style dietary pattern, were associated with an unfavorable CKM risk profile (e.g. higher levels of blood pressure, glycated hemoglobin, triglycerides, visceral fat, and lower eGFR levels). Adherence to healthier patterns was associated with a favorable risk profile (e.g. lower levels of total and LDL cholesterol, triglycerides, blood pressure, glycated hemoglobin, visceral fat, and higher levels of HDL cholesterol. The hPDI was most favorably associated with eight out of ten CKM markers investigated, showing additionally positive associations with eGFR. Overall, associations tended to be stronger for healthy than for unhealthy dietary patterns in relation to CKM markers. The observed associations were partially modified by sex, BMI, smoking habit and physical activity.

### Associations between dietary patterns and CKM risk markers

To date, most studies have examined the association of diet with cardiovascular, kidney and cardiometabolic markers separately. Given the growing evidence for the interlinked nature of the conditions associated with these sets of biomarkers, now grouped together as CKM, we assessed the association of dietary patterns comprehensively with markers of all three conditions, with the greater aim to evaluate synergies between various diets and CKM health. This approach aligns with the HEARTS 2.0, a World Health Organization’s (WHO) Global HEARTS Initiative, which aims to advance the HEARTS Clinical Pathway for CKM management by integrating evidence-based interventions into a unified care pathway, proposing also non-pharmacological interventions for CKM management, including diet [42].

We present here the main findings with discussion, together with a detailed comparison to literature provided in Supplementary Table S6) Our findings highlight the relevant role of predominantly plant-rich diets, specifically the hPDI, AHEI and DASH, in the management of blood lipids, which largely confirms previous evidence coming from observational studies and meta-analyses of randomized controlled trials [43–47]. Compared to omnivorous diets, plant-based diets are usually higher in poly-unsaturated fatty acids, while lower in saturated fatty acids, cholesterol and total fat. Lower fat consumption leads to reduced intestinal absorption of triglycerides and cholesterol, thus decreasing cholesterol containing lipoprotein particles in the blood [47].

We further found most healthy dietary patterns to be associated with lower blood pressure levels, with particularly strong associations observed with the DASH diet. Unhealthy patterns, except UPF, were associated with higher blood pressure levels. Our findings strongly align with previous literature including both observational studies and meta-analyses of randomized clinical trials, except for the MED diet and UPF [6, 41, 46, 48, 49]. Several mechanisms of the beneficial effects of plant-rich diets have been discussed. For example, flavonoid rich fruits and nitrate-rich vegetables are able to increase the plasma nitric oxide concentrations, which has been linked to improved endothelial function and decreased blood pressure. Moreover, plant-based foods might decrease blood pressure through decreased blood viscosity, modifications of both the renin–angiotensin and the sympathetic nervous system [6].

All healthy dietary patterns were associated with lower levels of glycemia, while uPDI was associated with higher levels, which confirms previous evidence coming from observational studies [44–46]. Various biological pathways linking plant-rich diets to lower T2DM risk have been discussed. Specifically, higher intake of dietary fiber and polyphenols and lower intake of saturated fats might impact weight control, inflammation, antioxidant levels, as well as gut microbiome composition, all factors shown to regulate insulin sensitivity [50–53].

We further observed the AHEI, DASH and Prudent style dietary pattern (RC2) to be associated with less visceral fat, while uPDI and the Western style dietary pattern (RC1) were associated with more visceral fat. No associations were observed with the other indices. These findings partially align with previous evidence, which includes observational studies as well as meta-analyses of observational studies and randomized controlled trials [46, 54, 55].

Regarding kidney health, our analysis showed that adherence to hPDI was associated with higher eGFR, while a Western style dietary pattern (RC1) and higher UPF consumption were associated with lower eGFR levels, which confirms previous evidence from observational studies investigating incident CKD and decline of kidney function in the general population [56–58], as well as in overweight/obese subjects with metabolic syndrome [59], and subjects with and without diabetes, with the latter reporting associations to being stronger in those with diabetes as compared to without [60]. Western style diets are usually characterized by high intake of processed and unprocessed meat. A high consumption of protein through animal sources has been associated with intraglomerular hypertension, hyperfiltration, and glomerular injury, which may contribute to proteinuria and long-term renal decline [61]. Moreover, Western style diets are typically high in added sugars and sweeteners, which have been associated with the risk of albuminuria, incident CKD, and reduced eGFR [62]. These effects may be mediated or exacerbated by metabolic conditions such as obesity [59], diabetes [60], and hypertension, as well as elevated uric acid levels, all of which are recognized risk factors for CKD [63].

Overall, our findings highlight the relevant role that different dietary patterns play in relation to CKM risk markers, suggesting that certain dietary patterns may be more strongly associated with specific outcomes. For example, while the DASH diet was confirmed to be most strongly associated with lower blood pressure levels, AHEI, hPDI and uPDI showed strongest associations with lipids. When it comes to overall CKM health, hPDI specifically captured favorable associations with the main CKM risk factors, including dyslipidemia, glycemia, blood pressure and kidney function. In contrast, the uPDI was associated with unfavorable levels of most CKM markers, including visceral fat, but not kidney function. These findings suggest that also the quality of plant-based foods plays a relevant role in CKM health. While these associations are statistically significant, the respective effect magnitudes were generally small and should be carefully interpreted in the context of their potential clinical relevance. Some of these estimates align in scale with previous findings in the literature [6, 44, 55] and represent meaningful differences in disease risk. Take SBP for instance, where we observed differences of up to – 1.97 mmHg between groups in the first and fourth quintiles of various dietary pattern scores, and research indicates a 53% increase in atherosclerotic cardiovascular disease risk with each 10 mmHg increase [64] or HDL, where we saw differences of up to 1.86 mg/dL and research indicates roughly 25% reduction in myocardial infarction risk with each 5 mg/dL decrease [65].

### Effect modification by sex, BMI, physical activity and smoking

To what extent the relationship between diet and disease varies by specific population characteristics has been less explored. In our investigation we found various associations between dietary patterns and CKM markers to be modified by sex, BMI, physical activity and smoking. Sex was a strong effect modifier, with the associations between dietary patterns, blood lipids and kidney function being stronger in males than in females. There is mounting evidence indicating that biological sex affects the response to diet-based interventions and its impact on disease development [66]. A recent systematic review and meta-analysis found associations between vegetarian diets and reductions in total cholesterol and LDL cholesterol to be stronger in males as compared to females [5], which aligns with our observations. Moreover, several studies have indicated a low-fat/high-carbohydrate diet to induce more improved lipid profiles in males [66]. Such sex-differences might be explained by sexual dimorphism having a significant impact on nutrient requirements and response, glucose, lipid, as well as cardiac energy metabolism and function, and disease susceptibility, which might bedriven by factors such as sex hormones, sex-specific gene expression, and body fat distribution [66, 67].

Moreover, associations between dietary patterns, lipid levels, and visceral fat tended to be stronger in individuals with overweight/obesity. Contrasting our findings, two other studies found a BMI < 25 kg/m^2^ to be associated with greater reductions in lipid levels when following plant-based diets [5, 47]. Obesity exerts adverse effects on cholesterol metabolism, given that the hepatic and intestinal cholesterol synthesis is known to increase in obese individuals [68]. This could explain the fact that plant-based diets typically have a smaller impact on their cholesterol levels in plasma [47].

We observed that only few associations between dietary patterns (DASH, Prudent and Western style diet) and CKM markers (SBP, visceral fat) were modified by smoking status. Other studies have stated that the beneficial impact of diet on cardiometabolic health might be eliminated by the adverse impact of smoking [69, 70]. Moreover, although smokers tend to have a lower BMI [71], which might be caused by suppressed appetite, inhibited food intake and increased metabolic rate [72], smoking increases the risk of central obesity through antiestrogenic effect by increasing the 2-hydroxylation of estradiol or inducing an imbalance in androgenic to estrogenic activity in smokers [71, 73].

Moreover, physical activity modified associations between diet and some CKM markers (HDL, visceral fat, UACR), which aligns with previous findings [74–76]. Although mechanisms between these conjoint effects are not fully understood, intervention studies have demonstrated improved lipid profiles and glycemic control with reductions in blood pressure, body weight, fat mass and visceral fat in participants following a combination of healthy diet and physical activity. Moreover, from a behavioral perspective higher physical activity might indirectly promote healthier eating habits [76]. 

Although our findings support previous evidence of the modifying role of various characteristics in the diet-disease relationship, our exploratory findings need cautious interpretation, as our results might be susceptible to residual confounding from lifestyle-related factors, as well as unbalanced sample sizes in the stratified analyses affecting the power of the analysis. Nevertheless, understanding potential heterogeneity in the diet-health relationship could help us to optimize dietary guidelines in the prevention of CKM and needs to be further investigated.

### Strengths and limitations

Strengths of our investigation were the large sample size and the use of a validated semi-quantitative FFQ, which allowed us to compare various dietary patterns. Detailed information on medical and disease history, as well as lifestyle, allowed for control of known confounders. Finally, the access to clinical and biochemical data allowed us to assess various CKM risk markers together.

Limitations are primarily the cross-sectional nature of the data, leading to exposure and outcome data being assessed at the same time, and the non-randomized, observational nature of the research. These aspects limit the possibility of giving a causal interpretation of the results and prevent the exclusion of reverse causation. To attenuate this risk, we adjusted our models for awareness of current health conditions relevant to the CKM markers. As it is not feasible to measure glomerular filtration rate (GFR) in population studies, kidney function could only be approximated via eGFR, estimated based on serum creatinine. Creatinine is sensitive to muscle mass and protein intake, which could have affected our results, especially those reflecting dietary patterns with high protein intake. While other kidney function markers such as Cystatin C were not available, the analysis of 12-h fasting plasma samples has been shown to mitigate the acute effect of meat consumption on creatinine levels, making it unlikely that high creatinine levels leading to low eGFR could have been due to recent meat consumption [77].

Dietary intake assessment was self-reported and limited to a single, 12-month recall, potentially resulting in recall bias. Moreover, in our analysis females had higher mean values of all healthy dietary patterns than males. Although females tend to have a better dietary quality than males, they also tend to more often overreport healthy and underreport unhealthy foods, as females are more prone to the social desirability bias [78]. Such potential misreporting might have attenuated the true associations between diet and CKM markers observed in our analysis.

In addition, the GA^2^LEN FFQ was not originally designed to record UPFs. Many products that fall under NOVA category 4 were not specifically surveyed and therefore had to be classified based on product descriptions. This retrospective classification might limit the accuracy of the UPF survey and introduce the possibility of misclassification for certain products. Consequently, the true UPF exposure in this sample may have been underestimated. These factors might partially explain our null results for UPF and most of the CKM risk markers, except eGFR.

Moreover, we unexpectedly observed only weak or null associations with the MED, despite the Mediterranean diet being widely considered one of the most health-promoting dietary patterns. Several methodological and population-specific factors may explain the lack of associations in our study. First, the MED score used does not differentiate between important food components, such as lean versus processed meat, or low versus high fat dairy, which may attenuate the scores ability to detect true health promoting associations, as was seen with the other healthy dietary scores. Second, the variance of MED adherence in our study population may be limited, reducing statistical power to detect associations. Given that diet–disease relationships were detectable for other scores in our data, the MED score may not have been sufficiently sensitive or discriminative in our specific context.

Furthermore, although we adjusted our models for the awareness of hypertension, diabetes and CKD, we cannot exclude the presence of residual confounding due to other CKM related factors not captured by our study that could have influenced dietary intake.

Finally, our study was conducted in a rural Alpine area within a homogenous study population, where dietary habits might differ from urban centers. This might affect generalizability of our findings, though we have shown that sex-age post-stratification weighting can be used to ensure comparability between estimates of risk factors and health outcomes of interest generated by the study and those drawn from administrative data from the reference population, indicating the potential utility of statistical adjustment to improve generalizability [20].

## Conclusion

We addressed a gap in the research on the association between dietary patterns and markers of both cardiometabolic and kidney health. Results indicate that following a healthy plant-based diet, that primarily consists of higher consumption of fruits, vegetables, whole grains, and lower consumption of unhealthy fats, refined grains and processed plant and animal foods, is associated with favorable marker levels across all CKM systems. These findings emphasize the importance of further distinguishing between healthy and unhealthy plant-based foods. Population aging combined with the spread of unhealthy lifestyles may be a reason behind the increasing prevalence of CKM syndrome. Therefore, early identification and comprehensive management are essential to reducing its burden, especially at the subclinical, general population level.

## Supplementary Information

Below is the link to the electronic supplementary material.

Supplementary Material 1

## Data Availability

Data and samples can be requested for clearly defined research activities via the CHRIS Portal ([https://chrisportal.eurac.edu/) ).
