# Supplementary material for: Associations between dietary patterns and cardiovascular, kidney and metabolic risk markers in the Cooperative Health Research in South Tyrol study
Source: Eur J Nutr. 2026 May 29;65(4):146. doi: 10.1007/s00394-026-03992-y (PMC13221343; doi:10.1007/s00394-026-03992-y)
Supplement: Supplementary file 1 — Supplementary Material 1 [file 394_2026_3992_MOESM1_ESM.docx]

**Supplementary Material**

Title: Associations between dietary patterns and cardiovascular-kidney and metabolic risk markers in the Cooperative Health Research in South Tyrol Study”

Journal: European Journal of Nutrition

Authors: Essi Hantikainen, PhD^1^, Sophie Berger, BSc^2^, Giulia Barbieri, PhD^1^, Peter P. Pramstaller, MD^1^, Vanessa Garcia-Larsen, PhD^3^, Martin Gögele, PhD^1^, Cristian Pattaro, PhD^1^, Rebecca Lundin, ScD^1^, Francisco S. Domingues, PhD^1^

Affiliations:

1 Institute for Biomedicine, Eurac Research, Bolzano, Italy

2 Department of Nutritional Sciences, University of Vienna, Vienna, Austria

3 Program in Human Nutrition, Department of International Health, Bloomberg School of Public Health at Johns Hopkins University, Baltimore, MD, United States

Corresponding authors:

Essi Hantikainen

[essimarjatta.hantikainen@eurac.edu](mailto:essimarjatta.hantikainen@eurac.edu)

Francisco S. Domingues

[francisco.domingues@eurac.edu](mailto:francisco.domingues@eurac.edu)

Table of content

[Supplementary Text S1. Estimation of dietary patterns 3](#_Toc229552889)

[Supplementary Table S1. Summary of Dietary Patterns, Components, and Scoring Methods. 7](#_Toc229552890)

[Supplementary Table S2. Categorization of food processing for the GA^2^LEN FFQ foods and beverages according to Monteiro et al. 2010. 8](#_Toc229552891)

[Supplementary Table S3. Study participants’ characteristics by sex-specific quartiles one (Q1) and four (Q4) for each dietary pattern. 9](#_Toc229552892)

[Supplementary Table S4. Associations between dietary patterns and cardiovascular-kidney-metabolic risk markers. 13](#_Toc229552893)

[Supplementary Table S5. Associations between dietary patterns and cardiovascular-kidney-metabolic risk markers presenting standardised effect sizes. 17](#_Toc229552894)

[Supplementary Table S6. Comparison of current findings to relevant literature on dietary patterns and cardiovascular-kidney-metabolic risk markers. 20](#_Toc229552895)

[Supplementary Figure S1. Factor loadings for the first two rotated components of the principal component analysis (RC1 and RC2). 24](#_Toc229552897)

[Supplementary Figure S2. Distribution of age by sex in the study sample. 25](#_Toc229552899)

[Supplementary Figure S3. Spearman correlations between dietary patterns. …………………………….26](#_Toc229552900)

[Supplementary Figure S4. Heatmap presenting effect sizes for the associations between dietary patterns, modeled continuously (per 1 standard deviation increment), and cardiovascular-kidney-metabolic risk markers. 27](#_Toc229552901)

[Supplementary Figure S5. Subgroup analysis of the associations between dietary patterns and cardiovascular-kidney-metabolic risk markers by sex (n_male_=3673, n_female_=4393). 28](#_Toc229552903)

[Supplementary Figure S6. Subgroup analysis of the associations between dietary patterns and cardiovascular-kidney-metabolic risk markers by BMI (n_<25kg/m2_=3825 , n_>=25kg/m2_=4224). 29](#_Toc229552904)

[Supplementary Figure S7. Subgroup analysis of the associations between dietary patterns and cardiovascular-kidney-metabolic risk markers by physical activity level (n_<median_=3738, n_>=median_=3717). 30](#_Toc229552905)

[Supplementary Figure S8. Subgroup analysis of the associations between dietary patterns and cardiovascular-kidney-metabolic risk markers by smoking status (n _never/former_=6617, n_current smokers_=1415). 31](#_Toc229552906)

## Supplementary Text S1. Estimation of dietary patterns

*Alternative Healthy Eating Index 2010 (AHEI)*

The AHEI scores 11 components of dietary intake (servings per day) from 0 to 10 (worst to best), with total score ranging between 0 and 110 (non-adherence to perfect adherence). A score of 10 (healthiest) is assigned to *higher* intakes of fruit, vegetables, whole grains, nuts and legumes, omega-3 fatty acids, polyunsaturated fatty acids (PUFA), *moderate consumption* of alcohol, and *lower* intake of sugar-sweetened beverages (SSB), red/processed meat, trans-fat, sodium and heavy consumption of alcohol. Individual food components were scored following the cut-off criteria by Chuive *et al.* from which the AHEI-2010 score was derived [1].

*Mediterranean diet*

The Mediterranean Diet Score ranks participants based on 9 components of dietary intake (grams per day) scored from 0 to 1 (worst to best) [2]. For the putative beneficial components, vegetables, fruit, legumes, cereals, and fish and the ratio of monounsaturated and polyunsaturated fatty acids to saturated fatty acids (referred to as fatty acids ratio), an intake at or above the sex-specific median is scored as one, and an intake below the sex-specific median is scored as zero. For the putative detrimental components, dairy and meat, reverse scoring is used. Consumption of alcohol from 10 to 50 g/d for men and from 5 to 25 g/d for women is scored one; otherwise, the score is assigned as zero. The component scores are summed to obtain an overall diet score that ranges from 0 to 9, with higher scores indicating better adherence to a Mediterranean diet.

*Dietary Approach to Stop Hypertension (DASH) diet*

Adherence to a DASH-style diet was evaluated using eight food components identified by Fung et al. [3] for their influence on hypertension risk. Relevant food groups from the FFQ, expressed in weekly servings, were classified as either healthy (fruits, vegetables, whole grains, low-fat dairy, nuts and legumes) or unhealthy (red and processed meats, sodium, and sugar-sweetened beverages, SSB). To account for sex-specific energy intake, we applied the nutrient residual method. Each component was then divided into sex-specific quintiles and scored from 1 to 5 for healthy items, and inversely from 5 to 1 for unhealthy ones. Given the generally low intake of SSB (IQR: 1–7 servings/week) compared to the US population where the score originated, we used sex-specific tertiles to rank this component from 3 to 1. The final DASH score was calculated by summing all ranks, yielding a total score between 8 and 36.

*Plant-based dietary indices*

For the plant-based dietary indices the FFQ items were categorized into eighteen food groups as outlined by Satja et al. and the consumption of these were expressed in servings/day [4]. After categorizing intake into quintiles, either positive or reverse scores were assigned. For the positively scored items participants with an intake above the highest quintile received a score of 5 and those below the lowest quintile intake received a score of 1, whereas reverse value was assigned for the reversely scored items. For each participant the individual scores of the 18 food groups were then summed up to create the final score. For the PDI, a positive score was assigned to the “healthy” and “less-healthy”/“unhealthy” plant foods, and a reverse score to the animal-based foods. For the hPDI, positive scores were assigned to the “healthy” plant foods, and a reverse score to the “less-healthy”/“unhealthy” plant foods and the animal-based foods. For the uPDI, a positive score was assigned to the “less-healthy”/“unhealthy” plant foods and a reverse score to the “healthy” plant foods and the animal-based foods.

*Ultra-processed foods (UPF)*

UPFs are characterized by a poor nutrient profile, being high in added sugar, saturated fatty acids (SFAs) and salt and low in fiber and micronutrients. The manufacturing of these products include technical and chemical modification, adding food additives such as colorings, flavorings and flavoring enhancers, emulsifiers and emulsifying salts, a variety of sugars and artificial sweeteners, thickeners, agents for foaming, anti-foaming, bulking, carbonating, gelling and glazing, modified oils and isolated proteins [5].

All foods and beverages from the GA^2^LEN FFQ were classified according to the NOVA food classification system developed by Monteiro et al. [6], and the UPF group was identified as the food items in the group IV of the NOVA system, which refers to items such as industrially produced breads, cakes, confectionery, cereals, margarines, soft drinks, juices, reconstituted meat products, frozen foods and ready-meals. Since alcoholic beverages are not categorized in the NOVA classification system, they were not included when assessing UPF consumption. UPF consumption was expressed in %-energy coming from total energy intake per day. The detailed classification of the foods and beverages of the FFQ according to the NOVA classification system is displayed in Supplementary Table S3.

*Principal component analysis*

To derive population specific dietary patterns, we first aggregated all 229 food items and beverages into 26 food groups according to similarities in nutrient profiles. Next, we applied principal component analysis with the orthogonal rotation procedure varimax to the predefined food groups [7,8] using the principal function from the R package psych [9]. Each obtained principal component, which we refer to as the dietary patterns, represents a linear combination of all food groups, which are weighted by their factor loadings. For the purpose of this analysis, we selected the first two rotated principal components (RCs), which explained 12% and 10% of the total variance, respectively. For all participants a score for each dietary pattern was estimated by multiplying the standardized consumption frequencies for each food group (mean=0, variance=1) with the respective factor loading. A higher score indicates a higher adherence to the corresponding dietary pattern. We selected a factor loading cut-off of 0.4 to describe the patterns. RC1 was characterized by higher consumption of animal-based foods (red- and processed meat, poultry, fish), dressings, refined grains, potato, sugar sweetened beverages, pizza, spirits and will be referred to as the *Western Style dietary pattern (Supplementary Figure S1)*. RC2 was characterized by foods such as vegetables, fruits, whole grains, fish, dairy, and vegetable fats and will be referred to as the *Prudent Style dietary pattern (Supplementary Figure S1)*.

References

1. Chiuve SE, Fung TT, Rimm EB, Hu FB, McCullough ML, Wang M, et al. Alternative Dietary Indices Both Strongly Predict Risk of Chronic Disease. J Nutr. 2012;142:1009–18. https://doi.org/10.3945/jn.111.157222

2. Trichopoulou A, Orfanos P, Norat T, Bueno-de-Mesquita B, Ocké MC, Peeters PH, et al. Modified Mediterranean diet and survival: EPIC-elderly prospective cohort study. BMJ. 2005;330:991. https://doi.org/10.1136/bmj.38415.644155.8F

3. Fung TT. Adherence to a DASH-Style Diet and Risk of Coronary Heart Disease and Stroke in Women. Arch Intern Med. 2008;168:713. https://doi.org/10.1001/archinte.168.7.713

4. Satija A, Bhupathiraju SN, Spiegelman D, Chiuve SE, Manson JE, Willett W, et al. Healthful and Unhealthful Plant-Based Diets and the Risk of Coronary Heart Disease in U.S. Adults. J Am Coll Cardiol. 2017;70:411–22. https://doi.org/10.1016/j.jacc.2017.05.047

5. Monteiro CA, Cannon G, Moubarac J-C, Levy RB, Louzada MLC, Jaime PC. The UN Decade of Nutrition, the NOVA food classification and the trouble with ultra-processing. Public Health Nutr. 2018;21:5–17. https://doi.org/10.1017/S1368980017000234

6. Monteiro CA, Levy RB, Claro RM, Castro IRRD, Cannon G. A new classification of foods based on the extent and purpose of their processing. Cad Saúde Pública. 2010;26:2039–49. https://doi.org/10.1590/S0102-311X2010001100005

7. Zhao J, Li Z, Gao Q, Zhao H, Chen S, Huang L, et al. A review of statistical methods for dietary pattern analysis. Nutr J. 2021;20:37. https://doi.org/10.1186/s12937-021-00692-7

8. Varraso R, Garcia-Aymerich J, Monier F, Le Moual N, De Batlle J, Miranda G, et al. Assessment of dietary patterns in nutritional epidemiology: principal component analysis compared with confirmatory factor analysis. Am J Clin Nutr. 2012;96:1079–92. https://doi.org/10.3945/ajcn.112.038109

9. William Revelle (2025). psych: Procedures for Psychological, Psychometric, and Personality Research. Northwestern University, Evanston, Illinois. R package version 2.5.3, https://CRAN.R-project.org/package=psych.

## Supplementary Table S1. Summary of Dietary Patterns, Components, and Scoring Methods.

| **Dietary Index** | **Healthy Components** | **Unhealthy Components** | **Scoring Method** | **Score Range*** |
| --- | --- | --- | --- | --- |
| **DASH**  (Fung et al. 2008) | Fruits, vegetables, whole grains, low-fat dairy, nuts, legumes | Red and processed meats, sodium, sugar-sweetened beverages (SSBs) | Sex-specific quintiles: scored 1–5 for healthy components, 5–1 for unhealthy. SSBs scored 3–1 using sex-specific tertiles. | 8–37 |
| **AHEI**  (Chiave et al. 2012) | Fruits, vegetables, whole grains, nuts, legumes, omega-3s, PUFA, moderate alcohol | SSBs, red/processed meat, trans fats, sodium, heavy alcohol | Perfect adherence = 10, non-adherence = 0. Intermediate adherence scored proportionally. | 0–110 |
| **MED**  (Trichopoulou et al. 2005) | Vegetables, fruits, legumes, cereals, fish, MUFA+PUFA/SFA ratio | Dairy, meat | Beneficial components scored 1 if intake ≥ sex-specific median, else 0. Detrimental components scored inversely. | 0–9 |
|  | Alcohol Exception | | Alcohol intake of 10–50 g/d for men and 5–25 g/d for women scored 1, otherwise 0 |  |
| **PDI**  (Satja et al. 2017) | All plant foods (healthy and less healthy) | Animal foods | Intake of each food group divided into quintiles; higher quintile = higher score for plant foods, lower score for animal foods | 18–90 |
| **hPDI**  (Satja et al. 2017) | Healthy plant foods (whole grains, fruits, vegetables, nuts, legumes, vegetable oils, tea/coffee) | Less healthy plant foods and animal foods | Higher intake of healthy plant foods = higher score; less healthy and animal foods scored inversely | 18–90 |
| **uPDI**  (Satja et al. 2017) | Less healthy plant foods (refined grains, sweets, sugary beverages, potatoes) | Healthy plant foods and animal foods | Higher intake of less healthy plant foods = higher score; healthy and animal foods scored inversely | 18–90 |
| ***** *Score range: minimum = no adherence, maximum = perfect adherence*  *DASH: Dietary Approach to Stop Hypertension, AHEI: Alternate Healthy Eating Index, MED: Mediterranean diet, PDI: Plant-based Diet Index, hPDI: healthy plant-based diet index, uPDI: unhealthy Plant-based Diet Index, PUFA: polyunsaturated fatty acids, MUFA: Monounsaturated fatty acids, SFA: Saturated fatty acids* | | | | |

## Supplementary Table S2. Categorization of food processing for the GA^2^LEN FFQ foods and beverages according to Monteiro et al. 2010.

| **NOVA Classification** | **Food item** |
| --- | --- |
| **Group 1: unprocessed and minimally processed foods** | Couscous, Polenta, Rice (white), Rice (brown), Peanuts (dry roasted), Cashew nuts, Red kidney beans, Lentils, Chickpeas, Green beans, Broad beans, Soya beans, Lettuce, Spinach, Swiss chard, Amaranth leaves , Okra, Tomatoes, Aubergine, Courgette, Green peppers, Cucumber, Carrots, Parsnip, Turnip, Globe artichoke, Radish, Beetroot, Celery, Coleslaw (retail), Baby sweetcorn, Asparagus, Mixed herbs (dried), Leeks, Mushrooms, Onions, Garlic, Cauliflower, Pumpkin, Brussels sprouts, Green peas, Broccoli, Cabbage, Gherkins (pickled), Ginger, New potatoes, Sweet potato, Apples, Pears, Avocado, Mangoes, Apricots, Nectarines, Peaches, Plums, Cherries, Rhubarb, Blueberries, Bananas, Melon, Grapes, Orange juice, Pineapple, Kiwi, Lemon juice, Oranges, Grapefruit, Raisins, Figs, Prunes, Persimmons, Black tea, Coffee, Herbal tea, Beef, Pork, Veal, Rabbit, Lamb, Turkey, Sheep tongue, Salmon, Cod, Cod roe, Mussels, Eggs, Milk (semi-skimmed, skimmed, whole, sheep/goat, lactose-free, buttermilk, kefir), Yogurt (plain, Greek), Sour cream |
| **Group 2: processed culinary or food industry ingredients** | White sugar, Honey, Sunflower oil, Olive oil, Rapeseed oil, Butter (spreadable), Lard, Flaxseed oil, Pumpkin seed oil, Vegetable oil |
| **Group 3: processed food products** | Brown bread, White bread, Rye crispbread, Naan bread, Chapatis, Rusks, Pasta (plain, wholewheat), Egg noodles, Rice noodles, Mashed potatoes, Baked potatoes, Potato cakes, Potato chips, Canned mandarin oranges, Canned fruit cocktail, Green olives (in brine), Fruit juice drinks (diluted), Chicken (grilled, fricassee), Smoked salmon, Salted cod, Canned tuna, Canned crab, Omelette, Parmesan, Brie, Gouda, Cottage cheese, Mozzarella, Quark, Cream, Tofu, Pizza (cheese and tomato), Capers |
| **Group 4: ultra-processed food products** | Breakfast cereal, Ravioli (canned), Loaf cake, Danish pastries, Swiss roll, Muffins, Doughnuts, Tiramisu, Biscuits, Crackers, Jam, Boiled sweets, Cereal bars, Fruit lollies, Chocolate bars, Milk chocolate, Baking fat/margarine, Reduced-fat spread, Gnocchi, Carbonated fruit drinks, Soft/isotonic drinks (regular and diet), Pork sausages, Pork belly, Boiled ham, Frankfurters, Bacon rashers, Smoked/cured poultry, Liver pâté, Condensed milk, Soya milk (fortified), Ice cream, French dressing, Mayonnaise, White sauce, Ketchup, Wholemeal biscuits, Jam-filled biscuits, Energy drinks, Mustard, Fish fingers, Hazelnut spread (Nutella) |

| Supplementary Table S3. Study participants’ characteristics by sex-specific quartiles one (Q1) and four (Q4) for each dietary pattern. | | | | | | | | |
| --- | --- | --- | --- | --- | --- | --- | --- | --- |
| **Characteristics**  (Score range) | **AHEI**  (0–110) | | **MED**  (0–9) | | **DASH**  (8–37) | | **PDI**  (18–90) | |
|  | Q1, n=2016 | Q4, n=2018 | Q1, n=2016 | Q4, n=2018 | Q1, n=2016 | Q4, n=2018 | Q1, n=2016 | Q4, n=2018 |
| *Distribution of energy-adjusted score by sex* |  |  |  |  |  |  |  |  |
| Male | <53.4 | >69.7 | <3.1 | >5.4 | <17.2 | >23.9 | <49.3 | >56.9 |
| Female | <59.2 | >75.7 | <3.0 | >5.3 | <20.6 | >27.1 | <48.8 | >56.2 |
| *Sex, n (%)* |  |  |  |  |  |  |  |  |
| Male | 918 (45.5) | 919 (45.5) | 918 (45.5) | 919 (45.5) | 918 (45.5) | 919 (45.5) | 918 (45.5) | 919 (45.5) |
| Female | 1098 (54.5) | 1099 (54.5) | 1098 (54.5) | 1099 (54.5) | 1098 (54.5) | 1099 (54.5) | 1098 (54.5) | 1099 (54.5) |
| *Age, years, mean (SD)* | 41.9 (16.0) | 47.9 (16.5) | 42.3 (15.8) | 47.9 (17.0) | 40.0 (16.0) | 49.5 (16.2) | 44.6 (17.1) | 46.8 (16.7) |
| *BMI, kg/m^2^, mean (SD)* | 26.0 (4.77) | 25.4 (4.23) | 26.0 (4.69) | 25.7 (4.53) | 25.7 (4.75) | 25.8 (4.63) | 26.2 (4.74) | 25.5 (4.38) |
| *Total energy intake, kcal/day, mean (SD)* | 1930 (605) | 1950 (563) | 1970 (635) | 1960 (537) | 1960 (577) | 1970 (599) | 1970 (674) | 1980 (539) |
| *Alcohol, g/day, mean (SD)* | 5.40 (9.73) | 7.26 (6.88) | 4.41 (7.16) | 8.11 (8.95) | 6.24 (8.43) | 5.68 (7.44) | 6.87 (9.23) | 5.65 (7.47) |
| *Physical activity, IPAQ score)*, n (%) | |  |  |  |  |  |  |  |
| Low | 387 (19.2) | 249 (12.3) | 345 (17.1) | 278 (13.8) | 396 (19.6) | 238 (11.8) | 382 (18.9) | 253 (12.5) |
| Moderate | 500 (24.8) | 548 (27.2) | 512 (25.4) | 558 (27.7) | 498 (24.7) | 560 (27.8) | 522 (25.9) | 533 (26.4) |
| High | 945 (46.9) | 1089 (54.0) | 985 (48.9) | 1041 (51.6) | 945 (46.9) | 1085 (53.8) | 949 (47.1) | 1081 (53.6) |
| *Education, n (%)* |  |  |  |  |  |  |  |  |
| Primary school | 183 (9.1) | 170 (8.4) | 189 (9.4) | 194 (9.6) | 166 (8.2) | 219 (10.9) | 204 (10.1) | 195 (9.7) |
| Lower secondary school | 401 (19.9) | 247 (12.2) | 358 (17.8) | 246 (12.2) | 375 (18.6) | 269 (13.3) | 354 (17.6) | 322 (16.0) |
| Vocational school | 878 (43.6) | 803 (39.8) | 872 (43.3) | 808 (40.0) | 859 (42.6) | 795 (39.4) | 821 (40.7) | 797 (39.5) |
| Upper secondary school | 423 (21.0) | 530 (26.3) | 434 (21.5) | 524 (26.0) | 472 (23.4) | 477 (23.6) | 466 (23.1) | 502 (24.9) |
| University or higher | 127 (6.3) | 266 (13.2) | 160 (7.9) | 242 (12.0) | 141 (7.0) | 255 (12.6) | 166 (8.2) | 198 (9.8) |
| *Special diet, n (%)* | 109 (5.4) | 176 (8.7) | 105 (5.2) | 173 (8.6) | 99 (4.9) | 199 (9.9) | 157 (7.8) | 127 (6.3) |
| *Smoking, n (%)* |  |  |  |  |  |  |  |  |
| Never | 1035 (51.3) | 1126 (55.8) | 1107 (54.9) | 1082 (53.6) | 1049 (52.0) | 1152 (57.1) | 1071 (53.1) | 1123 (55.6) |
| Former | 449 (22.3) | 664 (32.9) | 482 (23.9) | 659 (32.7) | 427 (21.2) | 646 (32.0) | 564 (28.0) | 559 (27.7) |
| Current | 525 (26.0) | 217 (10.8) | 419 (20.8) | 264 (13.1) | 534 (26.5) | 209 (10.4) | 370 (18.4) | 330 (16.4) |
| *Comorbidity, yes, n (%)* |  |  |  |  |  |  |  |  |
| Hypertension^a^ | 423 (21.0) | 469 (23.2) | 392 (19.4) | 506 (25.1) | 368 (18.3) | 533 (26.4) | 454 (22.5) | 467 (23.1) |
| Dyslipidemia | 321 (15.9) | 521 (25.8) | 368 (18.3) | 525 (26.0) | 295 (14.6) | 570 (28.2) | 425 (21.1) | 492 (24.4) |
| Diabetes mellitus^a^ | 35 (1.7) | 51 (2.5) | 39 (1.9) | 62 (3.1) | 31 (1.5) | 74 (3.7) | 71 (3.5) | 44 (2.2) |
| Kidney disease^b^ | 166 (8.2) | 180 (8.9) | 162 (8.0) | 202 (10.0) | 156 (7.7) | 197 (9.8) | 154 (7.6) | 192 (9.5) |

| **Characteristics**  (Score range) | **hPDI**  (18–90) | | **uPDI**  (18–90) | | **UPF**  (0-0.71)^c^ | | **RC1**  (-4.12-5.23)^c^ | | **RC2**  (-4.31-5.05)^c^ | |
| --- | --- | --- | --- | --- | --- | --- | --- | --- | --- | --- |
|  | Q1, n=2016 | Q4, n=2018 | Q1, n=2016 | Q4, n=2018 | Q1, n=2016 | Q4, n=2018 | Q1, n=2016 | Q4, n=2018 | Q1, n=2016 | Q4, n=2018 |
| *Distribution of energy-adjusted score by sex* |  |  |  |  |  |  |  |  |  |  |
| Male | <48.8 | >59.3 | <50.5 | >60.1 | <.18 | >0.31 | <-0.2 | >0.8 | <-0.8 | >0.1 |
| Female | >49.9 | >60.6 | <49.3 | >58.4 | <0.16 | >0.27 | <-0.7 | >0.1 | >-0.2 | >0.7 |
| *Sex, n (%)* |  |  |  |  |  |  |  |  |  |  |
| Male | 918 (45.5) | 919 (45.5) | 918 (45.5) | 919 (45.5) | 918 (45.5) | 919 (45.5) | 918 (45.5) | 919 (45.5) | 918 (45.5) | 919 (45.5) |
| Female | 1098 (54.5) | 1099 (54.5) | 1098 (54.5) | 1099 (54.5) | 1098 (54.5) | 1099 (54.5) | 1098 (54.5) | 1099 (54.5) | 1098 (54.5) | 1099 (54.5) |
| *Age, years, mean (SD)* | 38.7 (15.6) | 51.9 (15.7) | 48.5 (15.4) | 42.0 (17.4) | 51.1 (16.7) | 41.7 (16.0) | 53.6 (15.4) | 36.5 (15.0) | 41.6 (16.5) | 48.6 (16.4) |
| *BMI, kg/m^2^, mean (SD)* | 25.6 (4.60) | 26.1 (4.60) | 26.1 (4.55) | 25.7 (4.55) | 26.3 (4.56) | 25.5 (4.57) | 25.7 (4.28) | 25.9 (4.81) | 25.9 (4.66) | 25.9 (4.62) |
| *Total energy intake, kcal/day, mean (SD)* | 1970 (528) | 1970 (649) | 1980 (510) | 1960 (712) | 1790 (563) | 2140 (674) | 2090 (620) | 2070 (648) | 2070 (667) | 2050 (633) |
| *Alcohol, g/day, mean (SD)* | 5.49 (6.85) | 6.65 (8.92) | 6.15 (7.70) | 6.06 (8.65) | 7.36 (9.61) | 5.36 (7.42) | 5.29 (7.70) | 7.45 (9.03) | 7.83 (10.6) | 4.96 (6.72) |
| *Physical activity, IPAQ score)*, n (%) | |  |  |  |  |  |  |  |  |  |
| Low | 354 (17.6) | 254 (12.6) | 248 (12.3) | 394 (19.5) | 338 (16.8) | 329 (16.3) | 287 (14.2) | 337 (16.7) | 398 (19.7) | 225 (11.1) |
| Moderate | 544 (27.0) | 538 (26.7) | 518 (25.7) | 517 (25.6) | 545 (27.0) | 522 (25.9) | 559 (27.7) | 518 (25.7) | 494 (24.5) | 537 (26.6) |
| High | 953 (47.3) | 1086 (53.8) | 1099 (54.5) | 937 (46.4) | 985 (48.9) | 987 (48.9) | 1028 (51.0) | 994 (49.3) | 930 (46.1) | 1115 (55.3) |
| *Education, n (%)* |  |  |  |  |  |  |  |  |  |  |
| Primary school | 134 (6.6) | 262 (13.0) | 179 (8.9) | 214 (10.6) | 296 (14.7) | 348 (17.2) | 325 (16.1) | 93 (4.6) | 182 (9.0) | 199 (9.9) |
| Lower secondary school | 363 (18.0) | 303 (15.0) | 269 (13.3) | 408 (20.2) | 294 (14.6) | 163 (8.1) | 309 (15.3) | 346 (17.1) | 431 (21.4) | 285 (14.1) |
| Vocational school | 848 (42.1) | 832 (41.2) | 838 (41.6) | 802 (39.7) | 222 (11.0) | 149 (7.4) | 827 (41.0) | 806 (39.9) | 870 (43.2) | 834 (41.3) |
| Upper secondary school | 517 (25.6) | 412 (20.4) | 464 (23.0) | 468 (23.2) | 404 (20.0) | 476 (23.6) | 359 (17.8) | 603 (29.9) | 410 (20.3) | 450 (22.3) |
| University or higher | 148 (7.3) | 205 (10.2) | 263 (13.0) | 123 (6.1) | 796 (39.5) | 879 (43.6) | 191 (9.5) | 164 (8.1) | 119 (5.9) | 246 (12.2) |
| *Special diet, yes, n (%)* | 89 (4.4) | 200 (9.9) | 186 (9.2) | 113 (5.6) | 192 (9.5) | 124 (6.1) | 135 (6.7) | 142 (7.0) | 90 (4.5) | 211 (10.5) |
| *Smoking, n (%)* |  |  |  |  |  |  |  |  |  |  |
| Never | 1073 (53.2) | 1105 (54.8) | 1082 (53.7) | 1094 (54.2) | 1123 (55.7) | 1081 (53.6) | 1224 (60.7) | 982 (48.7) | 990 (49.1) | 1139 (56.4) |
| Former | 418 (20.7) | 688 (34.1) | 676 (33.5) | 416 (20.6) | 603 (29.9) | 487 (24.1) | 603 (29.9) | 446 (22.1) | 465 (23.1) | 640 (31.7) |
| Current | 519 (25.7) | 217 (10.8) | 251 (12.5) | 504 (25.0) | 278 (13.8) | 444 (22.0) | 178 (8.8) | 581 (28.8) | 557 (27.6) | 226 (11.2) |
| *Comorbidity, yes, n (%)* | |  |  |  |  |  |  |  |  |  |
| Hypertension^a^ | 348 (17.3) | 581 (28.8) | 494 (24.5) | 450 (22.3) | 613 (30.4) | 386 (19.1) | 587 (29.1) | 296 (14.7) | 379 (18.8) | 509 (25.2) |
| Dyslipidemia ^a^ | 287 (14.2) | 604 (29.9) | 519 (25.7) | 366 (18.1) | 581 (28.8) | 328 (16.3) | 598 (29.7) | 272 (13.5) | 305 (15.1) | 549 (27.2) |
| Diabetes mellitus^a^ | 22 (1.1) | 89 (4.4) | 74 (3.7) | 38 (1.9) | 76 (3.8) | 33 (1.6) | 60 (3.0) | 39 (1.9) | 35 (1.7) | 65 (3.2) |
| Kidney disease^b^ | 152 (7.5) | 220 (10.9) | 178 (8.8) | 170 (8.4) | 205 (10.2) | 170 (8.4) | 208 (10.3) | 120 (5.9) | 161 (8.0) | 201 (10.0) |
| *Q1:* quartile one*, Q4:* quartile four*, SD* standard deviation, *BMI* body mass index, *IPAQ* International Physical Activity Questionnaire, *DASH*: Dietary Approach to Stop Hypertension, *AHEI*: Alternate Healthy Eating Index, *MED*: Mediterranean diet, *PDI*: Plant-based Diet Index, *hPDI*: healthy plant-based diet index, *uPDI*: unhealthy Plant-based Diet Index, *RC1*: First rotated principal component (Western style dietary pattern), *RC2*: Second rotated principal component (Prudent style dietary pattern)  *^a^Comorbidity awareness was defined as self-reported presence of the condition or taking medication to treat the condition. ^b^Kidney disease was defined as self-reported presence of the condition. ^c^Score range values correspond to the actual minimum and maximum values observed in the study sample.* | | | | | | | | | | |

## **Supplementary Table S4. Associations between dietary patterns and cardiovascular-kidney-metabolic risk markers.**

Associations were obtained from multivariable linear regression models comparing quartiles Q2, Q3 and Q4 to reference quartile Q1. Models were adjusted for: age and sex, physical activity, education, smoking, special diet, BMI, awareness (medication or self-reported) of hypertension, diabetes, or lipid disturbance, and self-reported kidney disease, total energy intake and alcohol intake (g per day) for DASH, PDI, uPDI and hPDI. P: P-value after correction for multiple comparison using FDR. P-trend: P-value of the linear trend analysis without further corrections for multiple comparisons. DASH: Dietary Approach to Stop Hypertension, AHEI: Alternate Healthy Eating Index, MED: Mediterranean diet, PDI: Plant-based Diet Index, hPDI: healthy plant-based diet index, uPDI: unhealthy Plant-based Diet Index. RC1: First rotated principal component (Western style dietary pattern), RC2: second rotated principal component (Prudent style dietary pattern).

| CKM risk marker | Dietary pattern | Q2 vs Q1  Coef (95% CI) | Q3 vs Q1  Coef (95% CI) | Q4 vs Q1  Coef (95% CI) | P trend |
| --- | --- | --- | --- | --- | --- |
| Visceral fat | DASH | -0.17 (-0.28–-0.07), P=0.067 | -0.10 (-0.21–0.01), P=0.214 | -0.21 (-0.33–-0.10),  P=0.001 | 0.002 |
| Visceral fat | PDI | 0.02 (-0.09–0.12), P=0.879 | -0.02 (-0.13–0.08), P=0.768 | -0.09 (-0.20–0.02), P=0.168 | 0.070 |
| Visceral fat | uPDI | 0.07 (-0.04–0.17), P=0.579 | 0.14 (0.03–0.25), P=0.065 | 0.19 (0.08–0.30), P=0.003 | <0.001 |
| Visceral fat | hPDI | -0.07 (-0.17–0.04), P=0.579 | -0.06 (-0.17–0.05), P=0.457 | -0.07 (-0.18–0.04), P=0.313 | 0.245 |
| Visceral fat | MED | 0.03 (-0.08–0.13), P=0.824 | -0.02 (-0.13–0.08), P=0.768 | -0.08 (-0.18–0.03), P=0.268 | 0.107 |
| Visceral fat | AHEI | -0.01 (-0.12–0.10), P=0.942 | -0.12 (-0.23–-0.01), P=0.098 | -0.13 (-0.24–-0.02), P=0.041 | 0.004 |
| Visceral fat | RC1 | 0.16 (0.05–0.26), P=0.103 | 0.13 (0.02–0.24), P=0.085 | 0.13 (0.02–0.25), P=0.053 | 0.045 |
| Visceral fat | RC2 | -0.14 (-0.25–-0.03), P=0.136 | -0.14 (-0.25–-0.03), P=0.065 | -0.24 (-0.35–-0.13), P=<0.001 | <0.001 |
| Visceral fat | UPF | -0.01 (-0.12–0.09), P=0.918 | -0.02 (-0.13–0.09), P=0.833 | -0.05 (-0.16–0.07), P=0.506 | 0.418 |
| Total Cholesterol | DASH | 2.06 (-0.48–4.61), P=0.458 | -1.85 (-4.43–0.73), P=0.360 | -4.65 (-7.30–-2.01), P=0.003 | <0.001 |
| Total Cholesterol | PDI | 0.96 (-1.57–3.48), P=0.721 | -1.43 (-3.97–1.11), P=0.457 | -1.37 (-3.93–1.18), P=0.393 | 0.112 |
| Total Cholesterol | uPDI | 0.77 (-1.74–3.29), P=0.773 | 3.25 (0.70–5.81), P=0.071 | 0.63 (-1.97–3.23), P=0.681 | 0.301 |
| Total Cholesterol | hPDI | 0.29 (-2.25–2.82), P=0.927 | 2.28 (-0.31–4.86), P=0.226 | -5.02 (-7.67–-2.36), P=0.001 | 0.002 |
| Total Cholesterol | MED | 1.53 (-0.99–4.05), P=0.579 | 1.04 (-1.51–3.58), P=0.607 | -1.47 (-4.04–1.10), P=0.367 | 0.237 |
| Total Cholesterol | AHEI | 0.16 (-2.38–2.69), P=0.956 | -1.48 (-4.05–1.09), P=0.449 | -2.38 (-5.00–0.24), P=0.135 | 0.038 |
| Total Cholesterol | RC1 | 3.99 (1.43–6.54), P=0.067 | 3.23 (0.59–5.87), P=0.081 | 3.24 (0.47–6.01), P=0.053 | 0.045 |
| Total Cholesterol | RC2 | 1.03 (-1.55–3.60), P=0.699 | -1.62 (-4.21–0.98), P=0.408 | -5.37 (-7.99–-2.74), P=<0.001 | <0.001 |
| Total Cholesterol | UPF | 1.10 (-1.44–3.65), P=0.699 | 1.32 (-1.31–3.94), P=0.514 | 1.87 (-0.87–4.62), P=0.291 | 0.192 |
| SBP | DASH | -1.17 (-2.05–-0.29), P=0.136 | -1.25 (-2.15–-0.36), P=0.060 | -1.97 (-2.88–-1.05), P=<0.001 | <0.001 |
| SBP | PDI | -0.28 (-1.16–0.59), P=0.773 | -1.00 (-1.88–-0.13), P=0.090 | -0.57 (-1.45–0.32), P=0.314 | 0.090 |
| SBP | uPDI | 0.46 (-0.41–1.34), P=0.621 | 1.54 (0.66–2.42), P=0.019 | 1.89 (0.99–2.80), P=<0.001 | <0.001 |
| SBP | hPDI | -0.85 (-1.73–0.03), P=0.367 | -1.32 (-2.22–-0.43), P=0.048 | -1.61 (-2.53–-0.69), P=0.003 | <0.001 |
| SBP | MED | -0.11 (-0.98–0.76), P=0.918 | 0.35 (-0.53–1.23), P=0.607 | -0.05 (-0.94–0.84), P=0.915 | 0.826 |
| SBP | AHEI | -0.82 (-1.69–0.06), P=0.367 | -1.08 (-1.97–-0.19), P=0.081 | -1.57 (-2.47–-0.66), P=0.003 | <0.001 |
| SBP | RC1 | 0.30 (-0.59–1.18), P=0.766 | 0.96 (0.05–1.88), P=0.127 | 1.23 (0.27–2.19), P=0.033 | 0.005 |
| SBP | RC2 | -1.01 (-1.90–-0.12), P=0.260 | -1.23 (-2.13–-0.34), P=0.064 | -1.40 (-2.31–-0.48), P=0.008 | 0.003 |
| SBP | UPF | -0.25 (-1.13–0.63), P=0.775 | -0.30 (-1.21–0.60), P=0.669 | -1.04 (-1.99–-0.09), P=0.065 | 0.041 |
| ln(UACR) | DASH | -0.06 (-0.11–-0.00), P=0.317 | -0.03 (-0.09–0.02), P=0.408 | -0.07 (-0.12–-0.01), P=0.053 | 0.058 |
| ln(UACR) | PDI | -0.02 (-0.08–0.03), P=0.699 | 0.01 (-0.05–0.06), P=0.833 | -0.02 (-0.08–0.03), P=0.559 | 0.721 |
| ln(UACR) | uPDI | 0.01 (-0.04–0.07), P=0.870 | 0.03 (-0.03–0.08), P=0.503 | 0.05 (-0.00–0.11), P=0.109 | 0.048 |
| ln(UACR) | hPDI | -0.03 (-0.09–0.02), P=0.579 | -0.04 (-0.10–0.01), P=0.283 | -0.03 (-0.09–0.03), P=0.414 | 0.291 |
| ln(UACR) | MED | 0.00 (-0.05–0.05), P=0.984 | -0.01 (-0.07–0.04), P=0.768 | 0.01 (-0.05–0.06), P=0.832 | 0.926 |
| ln(UACR) | AHEI | -0.01 (-0.06–0.05), P=0.870 | -0.05 (-0.11–0.01), P=0.224 | -0.05 (-0.11–0.01), P=0.156 | 0.040 |
| ln(UACR) | RC1 | -0.03 (-0.08–0.03), P=0.621 | 0.04 (-0.02–0.10), P=0.387 | 0.02 (-0.04–0.08), P=0.594 | 0.187 |
| ln(UACR) | RC2 | -0.02 (-0.07–0.04), P=0.766 | -0.01 (-0.07–0.04), P=0.768 | -0.03 (-0.08–0.03), P=0.477 | 0.433 |
| ln(UACR) | UPF | 0.00 (-0.05–0.06), P=0.979 | 0.01 (-0.05–0.07), P=0.812 | -0.02 (-0.08–0.04), P=0.580 | 0.577 |
| ln(Triglycerides) | DASH | -0.01 (-0.04–0.02), P=0.699 | -0.00 (-0.03–0.03), P=0.833 | -0.03 (-0.06–0.00), P=0.106 | 0.100 |
| ln(Triglycerides) | PDI | 0.02 (-0.00–0.05), P=0.404 | 0.04 (0.01–0.07), P=0.064 | 0.06 (0.03–0.09), P=<0.001 | <0.001 |
| ln(Triglycerides) | uPDI | 0.02 (-0.01–0.05), P=0.459 | 0.06 (0.04–0.09), P=0.001 | 0.09 (0.06–0.12), P=<0.001 | <0.001 |
| ln(Triglycerides) | hPDI | -0.03 (-0.06–0.00), P=0.367 | -0.03 (-0.06–0.00), P=0.178 | -0.04 (-0.07–-0.01), P=0.033 | 0.017 |
| ln(Triglycerides) | MED | 0.03 (-0.00–0.06), P=0.367 | 0.05 (0.02–0.08), P=0.024 | 0.03 (0.00–0.06), P=0.053 | 0.010 |
| ln(Triglycerides) | AHEI | -0.01 (-0.04–0.02), P=0.754 | -0.01 (-0.04–0.02), P=0.679 | -0.03 (-0.06–-0.00), P=0.062 | 0.042 |
| ln(Triglycerides) | RC1 | 0.01 (-0.02–0.04), P=0.773 | 0.03 (0.00–0.06), P=0.098 | 0.04 (0.00–0.07), P=0.053 | 0.009 |
| ln(Triglycerides) | RC2 | -0.01 (-0.03–0.02), P=0.870 | -0.02 (-0.05–0.01), P=0.387 | -0.04 (-0.07–-0.01), P=0.027 | 0.006 |
| ln(Triglycerides) | UPF | 0.02 (-0.01–0.05), P=0.579 | 0.01 (-0.02–0.04), P=0.605 | 0.01 (-0.02–0.04), P=0.651 | 0.712 |
| ln(HbA1c) | DASH | -0.00 (-0.01–0.00), P=0.579 | -0.01 (-0.01–-0.00), P=0.081 | -0.01 (-0.01–-0.00), P=0.014 | 0.003 |
| ln(HbA1c) | PDI | 0.00 (-0.00–0.01), P=0.699 | -0.00 (-0.01–0.00), P=0.607 | -0.00 (-0.01–0.00), P=0.726 | 0.374 |
| ln(HbA1c) | uPDI | 0.00 (0.00–0.01), P=0.306 | 0.01 (0.00–0.01), P=0.081 | 0.01 (0.00–0.01), P=0.003 | <0.001 |
| ln(HbA1c) | hPDI | -0.00 (-0.01–0.00), P=0.475 | -0.00 (-0.01–0.00), P=0.408 | -0.01 (-0.01–-0.00), P=0.004 | 0.002 |
| ln(HbA1c) | MED | -0.00 (-0.01–-0.00), P=0.260 | -0.00 (-0.01–0.00), P=0.585 | -0.01 (-0.01–-0.00), P=<0.001 | 0.001 |
| ln(HbA1c) | AHEI | -0.00 (-0.01–0.00), P=0.579 | -0.01 (-0.01–-0.00), P=0.019 | -0.01 (-0.01–-0.00), P=0.003 | <0.001 |
| ln(HbA1c) | RC1 | 0.00 (-0.00–0.01), P=0.497 | 0.00 (-0.00–0.01), P=0.226 | 0.00 (-0.00–0.01), P=0.915 | 0.826 |
| ln(HbA1c) | RC2 | -0.00 (-0.01–0.00), P=0.699 | -0.00 (-0.01–0.00), P=0.819 | -0.01 (-0.01–-0.00), P=0.003 | 0.001 |
| ln(HbA1c) | UPF | 0.00 (-0.00–0.01), P=0.699 | 0.01 (0.00–0.01), P=0.065 | 0.00 (-0.00–0.01), P=0.393 | 0.122 |
| LDL | DASH | 2.65 (0.37–4.92), P=0.256 | -1.38 (-3.69–0.94), P=0.438 | -3.77 (-6.14–-1.40), P=0.005 | <0.001 |
| LDL | PDI | 0.66 (-1.60–2.92), P=0.775 | -1.98 (-4.25–0.29), P=0.226 | -1.30 (-3.59–0.99), P=0.367 | 0.075 |
| LDL | uPDI | 1.36 (-0.90–3.61), P=0.579 | 3.00 (0.71–5.29), P=0.065 | 0.29 (-2.04–2.63), P=0.832 | 0.500 |
| LDL | hPDI | 0.49 (-1.78–2.76), P=0.853 | 1.56 (-0.75–3.88), P=0.387 | -4.96 (-7.33–-2.58), P=<0.001 | <0.001 |
| LDL | MED | 0.42 (-1.83–2.68), P=0.870 | 0.31 (-1.97–2.58), P=0.833 | -2.11 (-4.42–0.19), P=0.132 | 0.082 |
| LDL | AHEI | -0.60 (-2.87–1.67), P=0.802 | -1.26 (-3.56–1.04), P=0.461 | -2.68 (-5.02–-0.33), P=0.053 | 0.022 |
| LDL | RC1 | 3.02 (0.73–5.31), P=0.136 | 1.56 (-0.81–3.92), P=0.395 | 1.60 (-0.87–4.08), P=0.313 | 0.401 |
| LDL | RC2 | 2.17 (-0.14–4.47), P=0.367 | -0.07 (-2.39–2.25), P=0.965 | -3.80 (-6.15–-1.45), P=0.005 | <0.001 |
| LDL | UPF | 0.99 (-1.29–3.27), P=0.699 | 0.81 (-1.54–3.16), P=0.663 | 1.40 (-1.06–3.86), P=0.367 | 0.317 |
| HDL | DASH | 0.23 (-0.55–1.00), P=0.775 | -0.10 (-0.89–0.68), P=0.833 | 0.54 (-0.26–1.35), P=0.297 | 0.322 |
| HDL | PDI | -0.05 (-0.82–0.71), P=0.955 | -0.20 (-0.97–0.57), P=0.768 | -0.79 (-1.57–-0.02), P=0.088 | 0.043 |
| HDL | uPDI | -0.66 (-1.43–0.10), P=0.404 | -1.09 (-1.87–-0.32), P=0.060 | -1.40 (-2.19–-0.61), P=0.003 | <0.001 |
| HDL | hPDI | 0.39 (-0.39–1.16), P=0.641 | 0.93 (0.14–1.72), P=0.083 | 1.01 (0.20–1.81),  P=0.037 | 0.006 |
| HDL | MED | 0.58 (-0.20–1.35), P=0.497 | 0.19 (-0.59–0.97), P=0.768 | 0.48 (-0.31–1.26), P=0.349 | 0.417 |
| HDL | AHEI | 1.25 (0.47–2.02), P=0.067 | 0.63 (-0.16–1.41), P=0.283 | 1.86 (1.06–2.66), P=<0.001 | <0.001 |
| HDL | RC1 | 0.02 (-0.76–0.81), P=0.979 | 0.30 (-0.51–1.11), P=0.639 | 0.26 (-0.58–1.11), P=0.610 | 0.435 |
| HDL | RC2 | -0.43 (-1.22–0.36), P=0.621 | -0.54 (-1.33–0.26), P=0.387 | -0.34 (-1.14–0.47), P=0.513 | 0.400 |
| HDL | UPF | 0.03 (-0.74–0.80), P=0.979 | 0.10 (-0.70–0.90), P=0.833 | 0.61 (-0.23–1.44), P=0.256 | 0.161 |
| eGFR | DASH | -0.59 (-1.31–0.14), P=0.458 | 0.25 (-0.48–0.99), P=0.663 | 0.30 (-0.45–1.05), P=0.532 | 0.144 |
| eGFR | PDI | 0.01 (-0.71–0.73), P=0.984 | 0.30 (-0.42–1.02), P=0.607 | 0.41 (-0.31–1.14), P=0.367 | 0.190 |
| eGFR | uPDI | -0.49 (-1.20–0.23), P=0.579 | -0.33 (-1.05–0.40), P=0.584 | -0.33 (-1.07–0.41), P=0.484 | 0.477 |
| eGFR | hPDI | 0.38 (-0.34–1.10), P=0.621 | 0.48 (-0.26–1.21), P=0.397 | 1.24 (0.49–2.00), P=0.005 | 0.002 |
| eGFR | MED | -0.40 (-1.11–0.32), P=0.621 | 0.22 (-0.50–0.94), P=0.693 | 0.21 (-0.52–0.94), P=0.638 | 0.287 |
| eGFR | AHEI | -0.35 (-1.07–0.37), P=0.657 | -0.15 (-0.88–0.58), P=0.787 | 0.25 (-0.49–1.00), P=0.589 | 0.420 |
| eGFR | RC1 | -0.22 (-0.95–0.50), P=0.773 | -1.23 (-1.97–-0.48), P=0.024 | -1.54 (-2.32–-0.76), P=<0.001 | <0.001 |
| eGFR | RC2 | -0.13 (-0.86–0.60), P=0.870 | 0.10 (-0.64–0.83), P=0.833 | 0.64 (-0.10–1.39), P=0.156 | 0.070 |
| eGFR | UPF | -0.75 (-1.48–-0.03), P=0.306 | -0.93 (-1.67–-0.19), P=0.076 | -1.05 (-1.82–-0.27), P=0.025 | 0.009 |
| DBP | DASH | -0.39 (-0.93–0.15), P=0.528 | -0.64 (-1.19–-0.09), P=0.089 | -1.18 (-1.74–-0.61), P<0.001 | <0.001 |
| DBP | PDI | -0.06 (-0.59–0.48), P=0.934 | 0.06 (-0.48–0.60), P=0.851 | -0.13 (-0.68–0.41), P=0.680 | 0.742 |
| DBP | uPDI | 0.13 (-0.41–0.67), P=0.824 | 0.42 (-0.12–0.97), P=0.296 | 0.64 (0.08–1.19), P=0.053 | 0.015 |
| DBP | hPDI | -0.35 (-0.90–0.19), P=0.579 | -0.32 (-0.87–0.23), P=0.449 | -0.93 (-1.49–-0.36), P=0.005 | 0.003 |
| DBP | MED | 0.28 (-0.26–0.81), P=0.632 | 0.19 (-0.35–0.73), P=0.663 | 0.06 (-0.49–0.60), P=0.861 | 0.928 |
| DBP | AHEI | -0.24 (-0.78–0.30), P=0.699 | -0.45 (-1.00–0.09), P=0.259 | -0.98 (-1.54–-0.42), P=0.003 | <0.001 |
| DBP | RC1 | 0.30 (-0.24–0.85), P=0.621 | 0.89 (0.33–1.45), P=0.030 | 0.19 (-0.41–0.78), P=0.610 | 0.218 |
| DBP | RC2 | -0.28 (-0.83–0.27), P=0.634 | -0.56 (-1.11–-0.00), P=0.149 | -0.90 (-1.46–-0.34), P=0.006 | 0.001 |
| DBP | UPF | 0.04 (-0.50–0.58), P=0.955 | -0.01 (-0.57–0.55), P=0.981 | -0.24 (-0.82–0.35),  P=0.530 | 0.426 |

## **Supplementary Table S5. Associations between dietary patterns and cardiovascular-kidney-metabolic risk markers presenting standardised effect sizes.**

Associations were obtained from multivariable linear regression models after scaling the CKM risk markers using z-standardisation and with dietary patterns analyzed continuously (per 1 standard deviation increment) and as quartiles (only Q4 vs Q1 is displayed in the table). Models were adjusted for: age and sex, physical activity, education, smoking, special diet, BMI, awareness (medication or self-reported) of hypertension, diabetes, or lipid disturbance, and self-reported kidney disease, total energy intake and alcohol intake (g per day) for DASH, PDI, uPDI and hPDI. P: P-value after correction for multiple comparison using FDR. DASH: Dietary Approach to Stop Hypertension, AHEI: Alternate Healthy Eating Index, MED: Mediterranean diet, PDI: Plant-based Diet Index, hPDI: healthy plant-based diet index, uPDI: unhealthy Plant-based Diet Index. RC1: First rotated principal component (Western style dietary pattern), RC2: second rotated principal component (Prudent style dietary pattern).

| **CKM risk marker** | **Dietary pattern** | **Continuous**  **(1 standard deviation increment)**  **Coef (95% CI)** | **Q4 vs Q1**  **Coef (95% CI)** |
| --- | --- | --- | --- |
| DBP | AHEI | -0.040 (-0.061, -0.018); P = 0.001 | -0.103 (-0.161, -0.044); P = 0.003 |
| DBP | DASH | -0.038 (-0.060, -0.015); P = 0.003 | -0.124 (-0.183, -0.064); P = <0.001 |
| DBP | hPDI | -0.035 (-0.056, -0.014); P = 0.004 | -0.097 (-0.157, -0.038); P = 0.005 |
| DBP | MED | -0.000 (-0.021, 0.020); P = 0.981 | 0.006 (-0.052, 0.063); P = 0.861 |
| DBP | PDI | 0.004 (-0.017, 0.024); P = 0.777 | -0.014 (-0.071, 0.043); P = 0.680 |
| DBP | RC1 | 0.013 (-0.012, 0.037); P = 0.405 | 0.019 (-0.043, 0.081); P = 0.610 |
| DBP | RC2 | -0.035 (-0.057, -0.013); P = 0.006 | -0.094 (-0.153, -0.035); P = 0.006 |
| DBP | uPDI | 0.034 (0.013, 0.055); P = 0.004 | 0.067 (0.008, 0.125); P = 0.053 |
| DBP | UPF | -0.008 (-0.030, 0.015); P = 0.578 | -0.025 (-0.086, 0.037); P = 0.530 |
| eGFR | AHEI | 0.006 (-0.012, 0.023); P = 0.590 | 0.016 (-0.031, 0.063); P = 0.589 |
| eGFR | DASH | 0.013 (-0.006, 0.031); P = 0.242 | 0.019 (-0.029, 0.067); P = 0.532 |
| eGFR | hPDI | 0.033 (0.016, 0.050); P = <0.001 | 0.079 (0.031, 0.127); P = 0.005 |
| eGFR | MED | 0.006 (-0.010, 0.023); P = 0.536 | 0.013 (-0.033, 0.059); P = 0.638 |
| eGFR | PDI | 0.014 (-0.003, 0.030); P = 0.165 | 0.026 (-0.020, 0.072); P = 0.367 |
| eGFR | RC1 | -0.062 (-0.081, -0.042); P = <0.001 | -0.098 (-0.147, -0.048); P = <0.001 |
| eGFR | RC2 | 0.016 (-0.002, 0.034); P = 0.152 | 0.041 (-0.006, 0.088); P = 0.156 |
| eGFR | uPDI | -0.012 (-0.028, 0.005); P = 0.242 | -0.021 (-0.068, 0.026); P = 0.484 |
| eGFR | UPF | -0.017 (-0.035, 0.001); P = 0.108 | -0.066 (-0.116, -0.017); P = 0.025 |
| HDL | AHEI | 0.043 (0.022, 0.065); P = <0.001 | 0.139 (0.079, 0.199); P = <0.001 |
| HDL | DASH | 0.019 (-0.004, 0.041); P = 0.174 | 0.040 (-0.020, 0.100); P = 0.297 |
| HDL | hPDI | 0.028 (0.006, 0.049); P = 0.028 | 0.075 (0.015, 0.136); P = 0.037 |
| HDL | MED | 0.012 (-0.009, 0.033); P = 0.375 | 0.036 (-0.023, 0.094); P = 0.349 |
| HDL | PDI | -0.023 (-0.043, -0.002); P = 0.062 | -0.059 (-0.117, -0.001); P = 0.088 |
| HDL | RC1 | 0.018 (-0.007, 0.042); P = 0.235 | 0.020 (-0.044, 0.083); P = 0.610 |
| HDL | RC2 | -0.010 (-0.033, 0.013); P = 0.499 | -0.025 (-0.085, 0.035); P = 0.513 |
| HDL | uPDI | -0.047 (-0.068, -0.026); P = <0.001 | -0.105 (-0.164, -0.046); P = 0.003 |
| HDL | UPF | 0.020 (-0.003, 0.042); P = 0.152 | 0.045 (-0.017, 0.108); P = 0.256 |
| LDL | AHEI | -0.034 (-0.057, -0.011); P = 0.011 | -0.073 (-0.136, -0.009); P = 0.053 |
| LDL | DASH | -0.052 (-0.076, -0.028); P = <0.001 | -0.102 (-0.167, -0.038); P = 0.006 |
| LDL | hPDI | -0.046 (-0.069, -0.023); P = <0.001 | -0.134 (-0.199, -0.070); P = <0.001 |
| LDL | MED | -0.025 (-0.047, -0.003); P = 0.054 | -0.057 (-0.120, 0.005); P = 0.132 |
| LDL | PDI | -0.017 (-0.039, 0.005); P = 0.207 | -0.035 (-0.097, 0.027); P = 0.367 |
| LDL | RC1 | 0.009 (-0.017, 0.035); P = 0.572 | 0.043 (-0.024, 0.111); P = 0.313 |
| LDL | RC2 | -0.050 (-0.075, -0.026); P = <0.001 | -0.103 (-0.167, -0.039); P = 0.005 |
| LDL | uPDI | 0.004 (-0.018, 0.027); P = 0.749 | 0.008 (-0.055, 0.071); P = 0.832 |
| LDL | UPF | 0.010 (-0.014, 0.035); P = 0.499 | 0.038 (-0.029, 0.105); P = 0.367 |
| log(HbA1c) | AHEI | -0.048 (-0.069, -0.027); P = <0.001 | -0.098 (-0.155, -0.041); P = 0.003 |
| log(HbA1c) | DASH | -0.042 (-0.064, -0.020); P = <0.001 | -0.083 (-0.141, -0.026); P = 0.014 |
| log(HbA1c) | hPDI | -0.035 (-0.056, -0.014); P = 0.003 | -0.097 (-0.155, -0.040); P = 0.004 |
| log(HbA1c) | MED | -0.032 (-0.052, -0.012); P = 0.005 | -0.110 (-0.166, -0.054); P = <0.001 |
| log(HbA1c) | PDI | -0.010 (-0.030, 0.010); P = 0.412 | -0.011 (-0.067, 0.044); P = 0.726 |
| log(HbA1c) | RC1 | -0.003 (-0.027, 0.020); P = 0.836 | 0.003 (-0.057, 0.063); P = 0.915 |
| log(HbA1c) | RC2 | -0.044 (-0.066, -0.022); P = <0.001 | -0.102 (-0.159, -0.045); P = 0.003 |
| log(HbA1c) | uPDI | 0.036 (0.015, 0.056); P = 0.002 | 0.100 (0.043, 0.156); P = 0.003 |
| log(HbA1c) | UPF | 0.010 (-0.012, 0.032); P = 0.467 | 0.032 (-0.027, 0.092); P = 0.393 |
| log(Triglycerides) | AHEI | -0.023 (-0.047, -0.000); P = 0.093 | -0.070 (-0.133, -0.007); P = 0.062 |
| log(Triglycerides) | DASH | -0.024 (-0.048, 0.000); P = 0.098 | -0.063 (-0.126, 0.001); P = 0.106 |
| log(Triglycerides) | hPDI | -0.031 (-0.054, -0.008); P = 0.018 | -0.082 (-0.146, -0.018); P = 0.033 |
| log(Triglycerides) | MED | 0.026 (0.004, 0.048); P = 0.047 | 0.071 (0.009, 0.133); P = 0.053 |
| log(Triglycerides) | PDI | 0.046 (0.024, 0.068); P = <0.001 | 0.127 (0.065, 0.188); P = <0.001 |
| log(Triglycerides) | RC1 | 0.050 (0.024, 0.076); P = <0.001 | 0.076 (0.010, 0.143); P = 0.053 |
| log(Triglycerides) | RC2 | -0.043 (-0.067, -0.019); P = 0.002 | -0.084 (-0.147, -0.020); P = 0.027 |
| log(Triglycerides) | uPDI | 0.076 (0.053, 0.098); P = <0.001 | 0.188 (0.126, 0.251); P = <0.001 |
| log(Triglycerides) | UPF | -0.007 (-0.032, 0.017); P = 0.620 | 0.018 (-0.048, 0.084); P = 0.651 |
| log(UACR) | AHEI | -0.018 (-0.043, 0.006); P = 0.207 | -0.058 (-0.124, 0.009); P = 0.156 |
| log(UACR) | DASH | -0.024 (-0.050, 0.001); P = 0.108 | -0.078 (-0.145, -0.011); P = 0.053 |
| log(UACR) | hPDI | -0.013 (-0.037, 0.011); P = 0.379 | -0.035 (-0.102, 0.033); P = 0.414 |
| log(UACR) | MED | 0.000 (-0.023, 0.023); P = 0.981 | 0.008 (-0.057, 0.073); P = 0.832 |
| log(UACR) | PDI | -0.010 (-0.033, 0.013); P = 0.499 | -0.024 (-0.088, 0.041); P = 0.559 |
| log(UACR) | RC1 | 0.006 (-0.022, 0.033); P = 0.745 | 0.023 (-0.047, 0.093); P = 0.594 |
| log(UACR) | RC2 | -0.002 (-0.028, 0.023); P = 0.879 | -0.031 (-0.097, 0.036); P = 0.477 |
| log(UACR) | uPDI | 0.023 (-0.001, 0.046); P = 0.108 | 0.064 (-0.002, 0.129); P = 0.109 |
| log(UACR) | UPF | -0.007 (-0.032, 0.019); P = 0.676 | -0.024 (-0.094, 0.045); P = 0.580 |
| SBP | AHEI | -0.035 (-0.055, -0.015); P = 0.002 | -0.093 (-0.146, -0.039); P = 0.003 |
| SBP | DASH | -0.033 (-0.053, -0.012); P = 0.005 | -0.116 (-0.171, -0.062); P = <0.001 |
| SBP | hPDI | -0.038 (-0.057, -0.018); P = <0.001 | -0.095 (-0.149, -0.041); P = 0.003 |
| SBP | MED | -0.002 (-0.020, 0.017); P = 0.879 | -0.003 (-0.056, 0.050); P = 0.915 |
| SBP | PDI | -0.009 (-0.028, 0.009); P = 0.410 | -0.034 (-0.086, 0.019); P = 0.314 |
| SBP | RC1 | 0.036 (0.014, 0.058); P = 0.004 | 0.073 (0.016, 0.129); P = 0.033 |
| SBP | RC2 | -0.031 (-0.052, -0.011); P = 0.007 | -0.083 (-0.136, -0.029); P = 0.008 |
| SBP | uPDI | 0.046 (0.027, 0.065); P = <0.001 | 0.112 (0.059, 0.165); P = <0.001 |
| SBP | UPF | -0.025 (-0.045, -0.004); P = 0.043 | -0.061 (-0.118, -0.005); P = 0.065 |
| Total cholesterol | AHEI | -0.029 (-0.052, -0.006); P = 0.028 | -0.057 (-0.119, 0.006); P = 0.135 |
| Total cholesterol | DASH | -0.052 (-0.076, -0.028); P = <0.001 | -0.111 (-0.174, -0.048); P = 0.003 |
| Total cholesterol | hPDI | -0.042 (-0.064, -0.019); P = 0.001 | -0.119 (-0.183, -0.056); P = 0.001 |
| Total cholesterol | MED | -0.017 (-0.039, 0.005); P = 0.195 | -0.035 (-0.096, 0.026); P = 0.367 |
| Total cholesterol | PDI | -0.015 (-0.037, 0.006); P = 0.235 | -0.033 (-0.093, 0.028); P = 0.393 |
| Total cholesterol | RC1 | 0.030 (0.004, 0.056); P = 0.047 | 0.077 (0.011, 0.143); P = 0.053 |
| Total cholesterol | RC2 | -0.060 (-0.084, -0.036); P = <0.001 | -0.128 (-0.190, -0.065); P = <0.001 |
| Total cholesterol | uPDI | 0.006 (-0.017, 0.028); P = 0.676 | 0.015 (-0.047, 0.077); P = 0.681 |
| Total cholesterol | UPF | 0.013 (-0.011, 0.037); P = 0.395 | 0.045 (-0.021, 0.110); P = 0.291 |
| Visceral fat | AHEI | -0.018 (-0.027, -0.009); P = <0.001 | -0.031 (-0.057, -0.006); P = 0.041 |
| Visceral fat | DASH | -0.016 (-0.026, -0.007); P = 0.003 | -0.050 (-0.075, -0.024); P = 0.001 |
| Visceral fat | hPDI | -0.010 (-0.019, -0.001); P = 0.073 | -0.017 (-0.043, 0.009); P = 0.313 |
| Visceral fat | MED | -0.007 (-0.016, 0.002); P = 0.169 | -0.018 (-0.043, 0.007); P = 0.268 |
| Visceral fat | PDI | -0.006 (-0.015, 0.002); P = 0.233 | -0.021 (-0.046, 0.004); P = 0.168 |
| Visceral fat | RC1 | 0.019 (0.008, 0.029); P = 0.002 | 0.031 (0.004, 0.058); P = 0.053 |
| Visceral fat | RC2 | -0.019 (-0.029, -0.009); P = <0.001 | -0.055 (-0.081, -0.030); P = <0.001 |
| Visceral fat | uPDI | 0.016 (0.007, 0.025); P = 0.002 | 0.044 (0.019, 0.069); P = 0.003 |
| Visceral fat | UPF | -0.009 (-0.019, 0.001); P = 0.124 | -0.011 (-0.038, 0.015); P = 0.506 |

## **Supplementary Table S6.** Comparison of current findings to relevant literature on dietary patterns and cardiovascular-kidney-metabolic risk markers.

|  | |
| --- | --- |
| CKM risk marker | **Discussion of dietary indices with relevant literature** |
| Lipids | Consistent with previous literature [1,2], we found the DASH diet to be associated with lower total and LDL cholesterol, but not with HDL cholesterol or triglycerides. The beneficial associations observed for AHEI, a Prudent style dietary pattern, and hPDI, as well as the unfavourable associations found for uPDI and a Western style dietary pattern largely align with findings from several observational studies and meta-analyses of RCTs [3–7]. Contrasting previous studies, the MED index [8,9] was not associated with lipids. While associations between UPF and HDL cholesterol appear more consistent across studies, findings for LDL cholesterol and triglycerides vary depending on the population under study and UPF classification system [10,11]. |
| Blood pressure | Our findings confirm previous evidence linking the DASH [1,12], as well as uPDI and the hPDI to blood pressure profiles [6,7,13]. Effects have also been reported for the MED index [12], which does not align with our findings. Literature on AHEI and blood pressure is limited and mixed associations have been reported in few observational studies [14,15]. Previous findings on the relationship between UPF and blood pressure show no associations [11], which is confirmed by our results. |
| Glycemia | Our results align with previous research showing association of AHEI with lower insulin levels [16], DASH with lower HbA1c [2] and hPDI and uPDI with fasting glucose [6] and T2DM risk [17]. Contrasting the latter evidence, which was based on a recent systematic review and dose-response meta-analysis of 16 prospective studies, a cross-sectional analysis did not find evidence of association between hPDI, uPDI and diabetes[7]. We did not observe any associations with UPF, and prior evidence on their relation to glycemia or diabetes is inconsistent [11,18]. |
| Visceral fat | Two systematic reviews and meta-analyses found predominantly plant-based diets to be related to lower visceral fat [19,20]**,** waist circumference and other anthropometric measures [7,21], reporting beneficial associations with AHEI, DASH, hPDI and MED. Our results confirm evidence for AHEI and DASH, but not for hPDI and MED. UPF has previously been associated with increased risk of obesity, increased levels of visceral fat and higher waist circumference [11,18], which was not confirmed by our findings. |
| Kidney health | Our findings regarding hPDI, a Western style diet and UPF align with previous literature demonstrating that plant-rich diets are associated with better kidney outcomes [22], while diets high in animal-sourced protein and processed foods are linked to worse kidney health [23]. No associations were observed between the DASH, AHEI or the MED diet, which does not align with previous evidence associating these diets to incident CKD [24,25]. |

*References*

1. Siervo M, Lara J, Chowdhury S, Ashor A, Oggioni C, Mathers JC. Effects of the Dietary Approach to Stop Hypertension (DASH) diet on cardiovascular risk factors: a systematic review and meta-analysis. Br J Nutr. 2015;113:1–15. https://doi.org/10.1017/S0007114514003341

2. Chiavaroli L, Viguiliouk E, Nishi S, Blanco Mejia S, Rahelić D, Kahleová H, et al. DASH Dietary Pattern and Cardiometabolic Outcomes: An Umbrella Review of Systematic Reviews and Meta-Analyses. Nutrients. 2019;11:338. https://doi.org/10.3390/nu11020338

3. Drake I, Sonestedt E, Ericson U, Wallström P, Orho-Melander M. A Western dietary pattern is prospectively associated with cardio-metabolic traits and incidence of the metabolic syndrome. Br J Nutr. 2018;119:1168–76. https://doi.org/10.1017/S000711451800079X

4. Faraji H, Jamshidi S, Ferrie S, Azar PS. The Relationship between Healthy Eating Index and Lipid Profile in Healthy Individuals: A Systematic Review. Int J Prev Med [Internet]. 2024 [cited 2025 Dec 1];15. https://doi.org/10.4103/ijpvm.ijpvm_404_22

5. Huang Y, Li X, Zhang T, Zeng X, Li M, Li H, et al. Associations of healthful and unhealthful plant-based diets with plasma markers of cardiometabolic risk. Eur J Nutr. 2023;62:2567–79. https://doi.org/10.1007/s00394-023-03170-4

6. Wang XJ, Steur M, Kavousi M, Voortman T. Adherence to plant-based diets and long-term changes in cardiometabolic markers: a longitudinal analysis in a population-based cohort. Am J Clin Nutr. 2025;122:424–32. https://doi.org/10.1016/j.ajcnut.2025.05.012

7. Dickinson KM, Marchese LE, Livingstone KM. Plant-Based Diet Quality Is Associated with Cardiometabolic Health in Adults: A Cross-Sectional Analysis of the Australian Health Survey. Nutrients. 2025;17:1621. https://doi.org/10.3390/nu17101621

8. Papadaki A, Nolen-Doerr E, Mantzoros CS. The Effect of the Mediterranean Diet on Metabolic Health: A Systematic Review and Meta-Analysis of Controlled Trials in Adults. Nutrients. 2020;12:3342. https://doi.org/10.3390/nu12113342

9. Kastorini C-M, Milionis HJ, Esposito K, Giugliano D, Goudevenos JA, Panagiotakos DB. The Effect of Mediterranean Diet on Metabolic Syndrome and its Components. J Am Coll Cardiol. 2011;57:1299–313. https://doi.org/10.1016/j.jacc.2010.09.073

10. Donat-Vargas C, Sandoval-Insausti H, Rey-García J, Moreno-Franco B, Åkesson A, Banegas JR, et al. High Consumption of Ultra-Processed Food is Associated with Incident Dyslipidemia: A Prospective Study of Older Adults. J Nutr. 2021;151:2390–8. https://doi.org/10.1093/jn/nxab118

11. Pagliai G, Dinu M, Madarena MP, Bonaccio M, Iacoviello L, Sofi F. Consumption of ultra-processed foods and health status: a systematic review and meta-analysis. Br J Nutr. 2021;125:308–18. https://doi.org/10.1017/S0007114520002688

12. Gibbs J, Gaskin E, Ji C, Miller MA, Cappuccio FP. The effect of plant-based dietary patterns on blood pressure: a systematic review and meta-analysis of controlled intervention trials. J Hypertens. Ovid Technologies (Wolters Kluwer Health); 2021;39:23–37. https://doi.org/10.1097/hjh.0000000000002604

13. Kim H, Rebholz CM, Garcia-Larsen V, Steffen LM, Coresh J, Caulfield LE. Operational Differences in Plant-Based Diet Indices Affect the Ability to Detect Associations with Incident Hypertension in Middle-Aged US Adults. J Nutr. 2020;150:842–50. https://doi.org/10.1093/jn/nxz275

14. Mattei J, Sotres-Alvarez D, Daviglus ML, Gallo LC, Gellman M, Hu FB, et al. Diet Quality and Its Association with Cardiometabolic Risk Factors Vary by Hispanic and Latino Ethnic Background in the Hispanic Community Health Study/Study of Latinos. J Nutr. 2016;146:2035–44. https://doi.org/10.3945/jn.116.231209

15. Mertens E, Markey O, Geleijnse JM, Lovegrove JA, Givens DI. Adherence to a healthy diet in relation to cardiovascular incidence and risk markers: evidence from the Caerphilly Prospective Study. Eur J Nutr. 2018;57:1245–58. https://doi.org/10.1007/s00394-017-1408-0

16. AlEssa HB, Malik VS, Yuan C, Willett WC, Huang T, Hu FB, et al. Dietary patterns and cardiometabolic and endocrine plasma biomarkers in US women. Am J Clin Nutr. 2017;105:432–41. https://doi.org/10.3945/ajcn.116.143016

17. Nikparast A, Mirzaei P, Tadayoni ZS, Asghari G. The Association Between Overall, Healthy, and Unhealthy Plant-Based Diet Index and Risk of Prediabetes and Type 2 Diabetes Mellitus: A Systematic Review and Dose-Response Meta-Analysis of Prospective Studies. Nutr Rev. 2025;83:e157–77. https://doi.org/10.1093/nutrit/nuae049

18. Mambrini SP, Menichetti F, Ravella S, Pellizzari M, De Amicis R, Foppiani A, et al. Ultra-Processed Food Consumption and Incidence of Obesity and Cardiometabolic Risk Factors in Adults: A Systematic Review of Prospective Studies. Nutrients. 2023;15:2583. https://doi.org/10.3390/nu15112583

19. Thimm A, Maskarinec G, Guillermo C, Nimptsch K, Pischon T. A systematic review of observational studies on the association between diet quality patterns and visceral adipose tissue. Br J Nutr. 2024;132:1530–41. https://doi.org/10.1017/S000711452400179X

20. Vij V, Deshmukh K, Vijayageetha M, Goyal C, Gumashta J, Gandhi AP. Effect of Predominantly Plant‐Based Diets on Visceral Fat: A Systematic Review and Meta‐Analysis. J Hum Nutr Diet. 2025;38:e70055. https://doi.org/10.1111/jhn.70055

21. Chen Z, Schoufour JD, Rivadeneira F, Lamballais S, Ikram MA, Franco OH, et al. Plant-based Diet and Adiposity Over Time in a Middle-aged and Elderly Population: The Rotterdam Study. Epidemiology. 2019;30:303–10. https://doi.org/10.1097/EDE.0000000000000961

22. Kim H, Caulfield LE, Garcia-Larsen V, Steffen LM, Grams ME, Coresh J, et al. Plant-Based Diets and Incident CKD and Kidney Function. Clin J Am Soc Nephrol. 2019;14:682–91. https://doi.org/10.2215/CJN.12391018

23. Hojjati Kermani MA, Awlqadr FH, Talebi S, Mehrabani S, Ghoreishy SM, Wong A, et al. Ultra-processed foods and risk of declined renal function: a dose–response meta-analysis of 786,216 participants. J Health Popul Nutr. 2025;44:79. https://doi.org/10.1186/s41043-025-00799-1

24. Mozaffari H, Ajabshir S, Alizadeh S. Dietary Approaches to Stop Hypertension and risk of chronic kidney disease: A systematic review and meta-analysis of observational studies. Clin Nutr. 2020;39:2035–44. https://doi.org/10.1016/j.clnu.2019.10.004

25. Hu EA, Steffen LM, Grams ME, Crews DC, Coresh J, Appel LJ, et al. Dietary patterns and risk of incident chronic kidney disease: the Atherosclerosis Risk in Communities study. Am J Clin Nutr. 2019;110:713–21. https://doi.org/10.1093/ajcn/nqz146

##
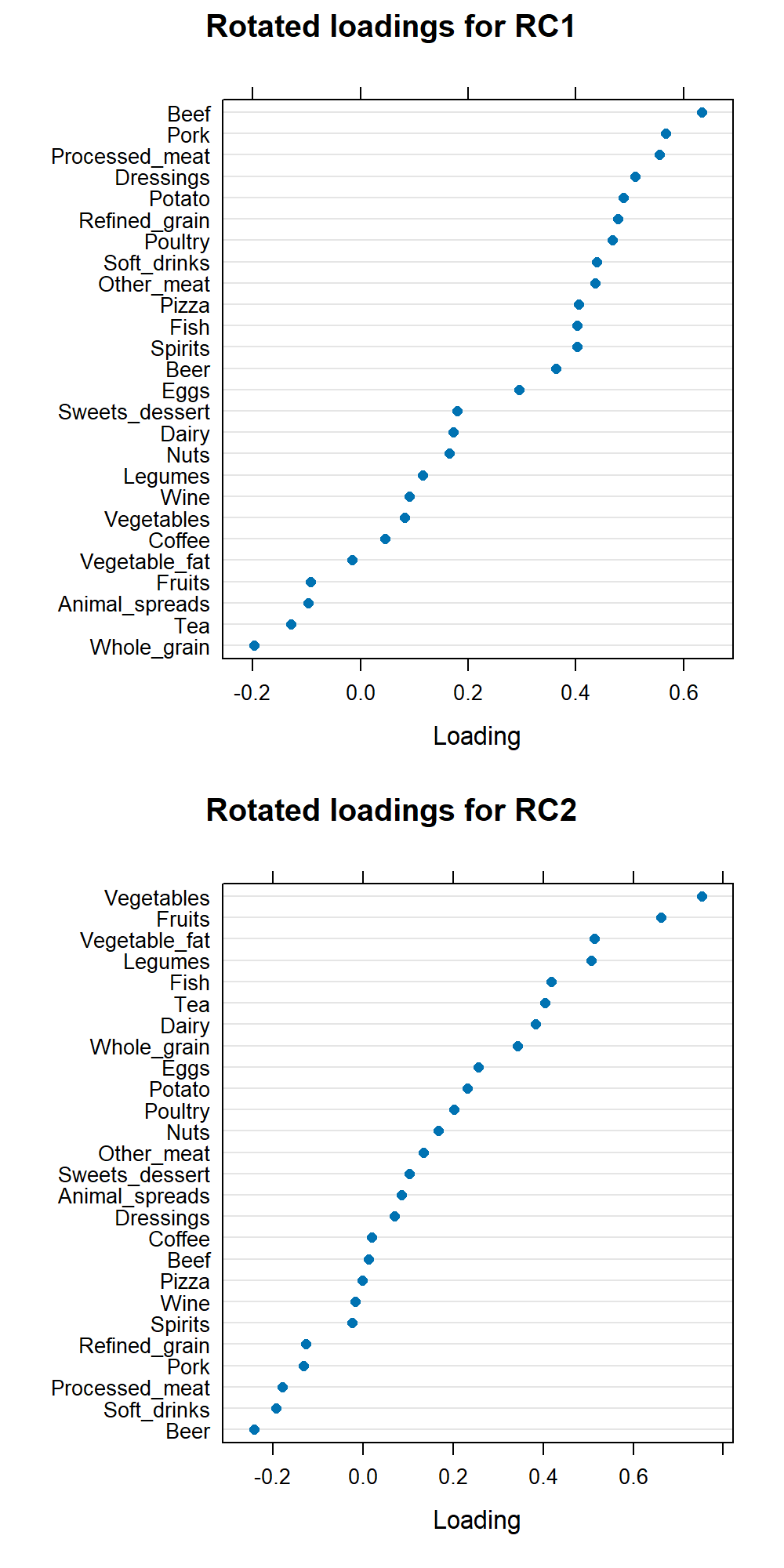


## **Supplementary Figure S1. Factor loadings for the first two rotated components of the principal component analysis (RC1 and RC2).**

## RC1: First rotated principal component (Western style dietary pattern), RC2: second rotated principal component (Prudent style dietary pattern).


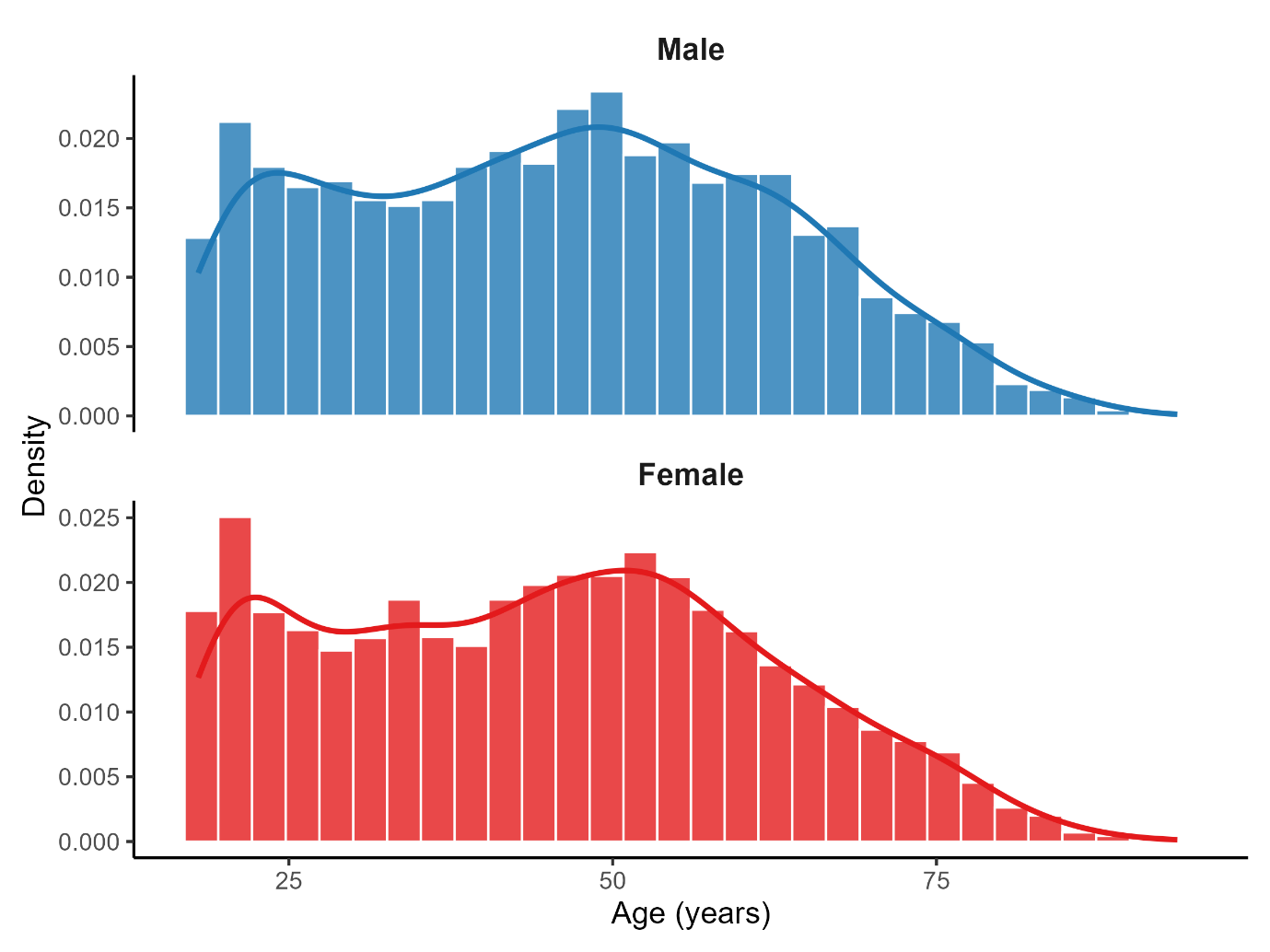


## Supplementary Figure S2. Distribution of age by sex in the study **sample**.

Sex‑stratified histograms with overlaid kernel density curves illustrate the age distribution among male and female participants.


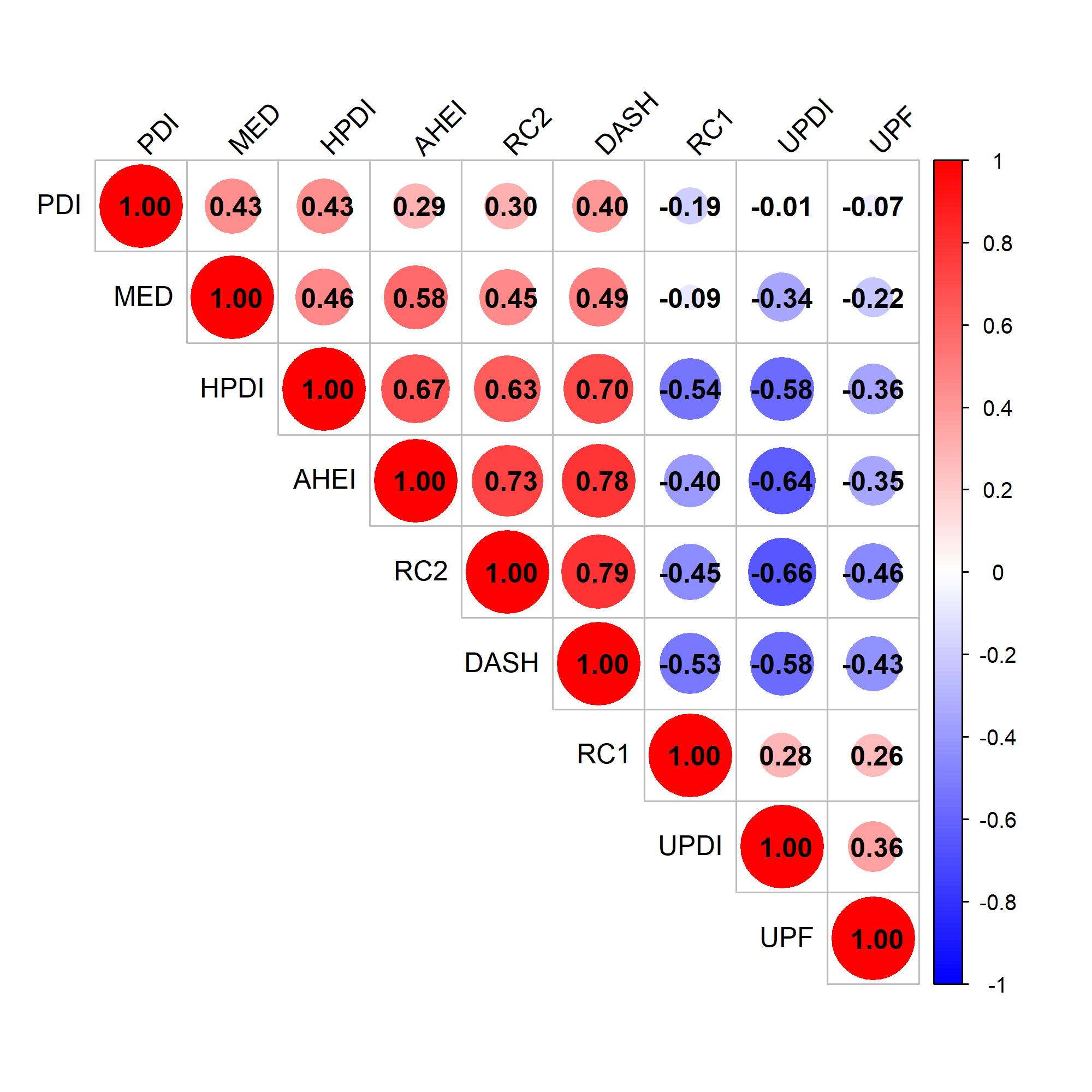


## **Supplementary Figure S3. Spearman correlations between dietary patterns.** All dietary indices were adjusted for total energy intake prior to the analysis, except UPF**.** DASH: Dietary Approach to Stop Hypertension, AHEI: Alternate Healthy Eating Index, MED: Mediterranean diet, PDI: Plant-based Diet Index, hPDI: healthy plant-based diet index, uPDI: unhealthy Plant-based Diet Index. RC1: First rotated principal component (Western style dietary pattern), RC2: second rotated principal component (Prudent style dietary pattern).


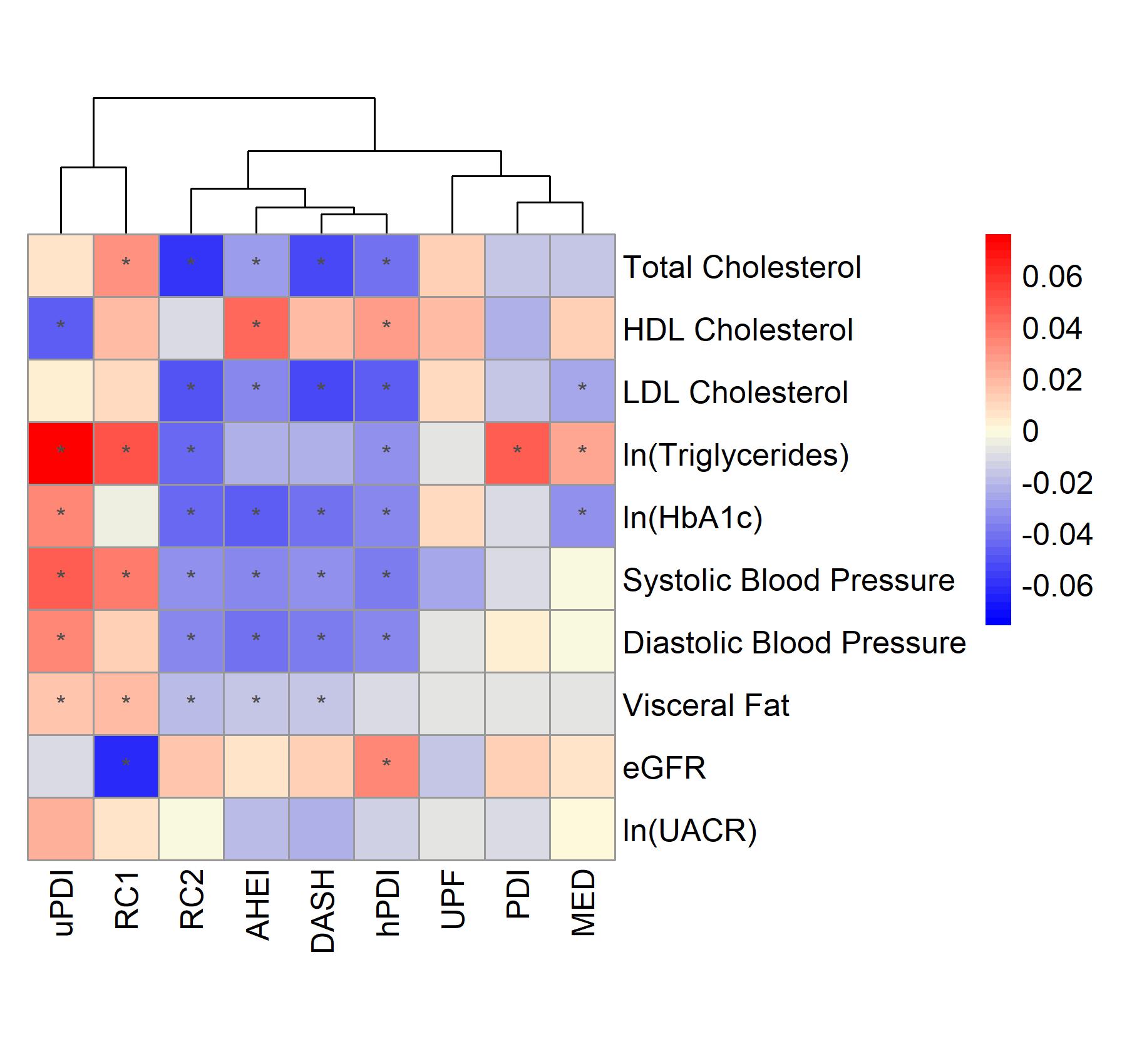


## **Supplementary Figure S4. Heatmap presenting effect sizes for the associations between dietary patterns, modeled continuously (per 1 standard deviation increment), and cardiovascular-kidney-metabolic risk markers.**

Associations were obtained from multivariable adjusted linear regression models after scaling both, the dietary patterns and the CKM risk markers using z-standardization. Linear regression models were adjusted for: age and sex, physical activity, education, smoking, special diet, BMI, medication or self-reported of hypertension, diabetes, or lipid disturbance, and self-reported kidney disease, total energy intake and alcohol intake for DASH, PDI, uPDI and hPDI. *: significant associations after correction for multiple comparison using FDR. DASH: Dietary Approach to Stop Hypertension, AHEI: Alternate Healthy Eating Index, MED: Mediterranean diet, PDI: Plant-based Diet Index, hPDI: healthy plant-based diet index, uPDI: unhealthy Plant-based Diet Index. RC1: First rotated principal component (Western style dietary pattern), RC2: second rotated principal component (Prudent style dietary pattern).

##
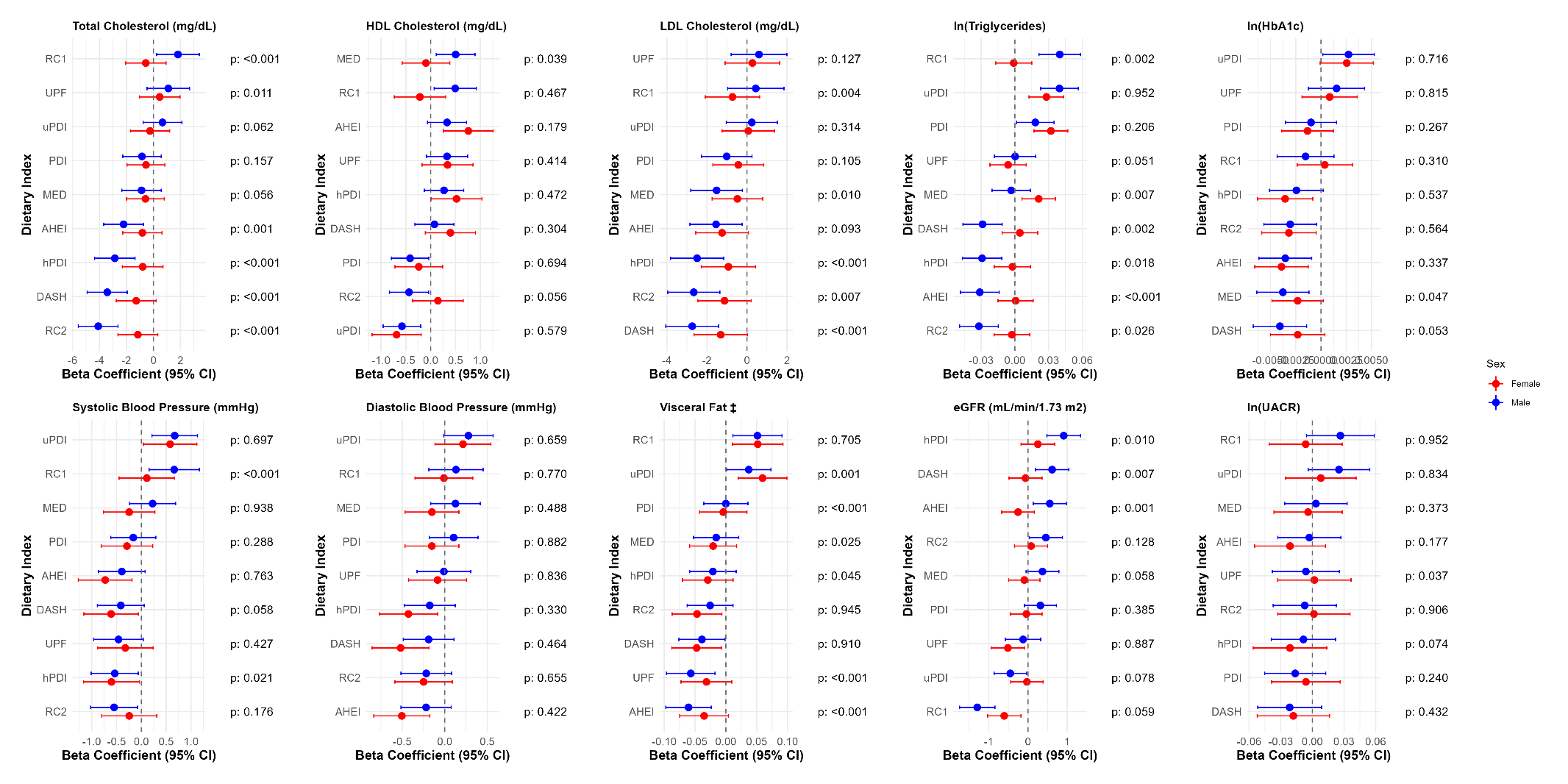


## Supplementary Figure S5. Subgroup analysis of the associations between dietary patterns and cardiovascular-kidney-metabolic risk markers by sex (n_male_=3673, n_female_=4393).

The forest plots show the beta-coefficients from the multivariable adjusted linear regression models for each of the dietary patterns analyzed continuously per 1 standard deviation increment (visually represented by the centers of the error bars), the 95% confidence intervals (visually represented by the error bars). The p‑value (p) shown originates from a separate interaction model and indicates the statistical significance of the interaction term. ‡ Visceral Fat Level index (range 1–30).


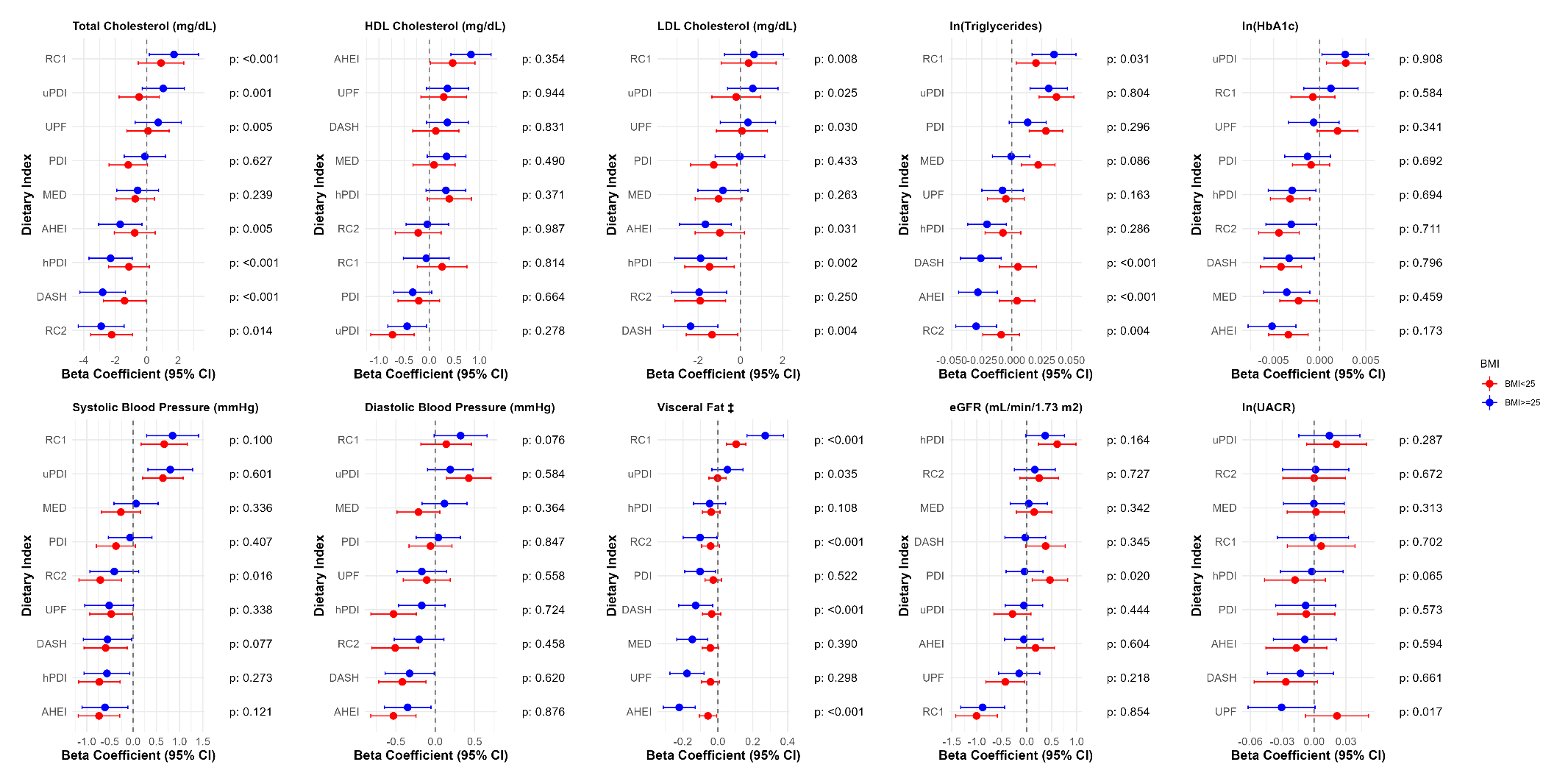


## Supplementary Figure S6. Subgroup analysis of the associations between dietary patterns and cardiovascular-kidney-metabolic risk markers by BMI (n_<25kg/m2_=3825 , n_>=25kg/m2_=4224).

The forest plots show the beta-coefficients from the multivariable adjusted linear regression models for each of the dietary patterns analyzed continuously per 1 standard deviation increment (visually represented by the centers of the error bars), the 95% confidence intervals (visually represented by the error bars). The p‑value (p) shown originates from a separate interaction model and indicates the statistical significance of the interaction term. ‡ Visceral Fat Level index (range 1–30).


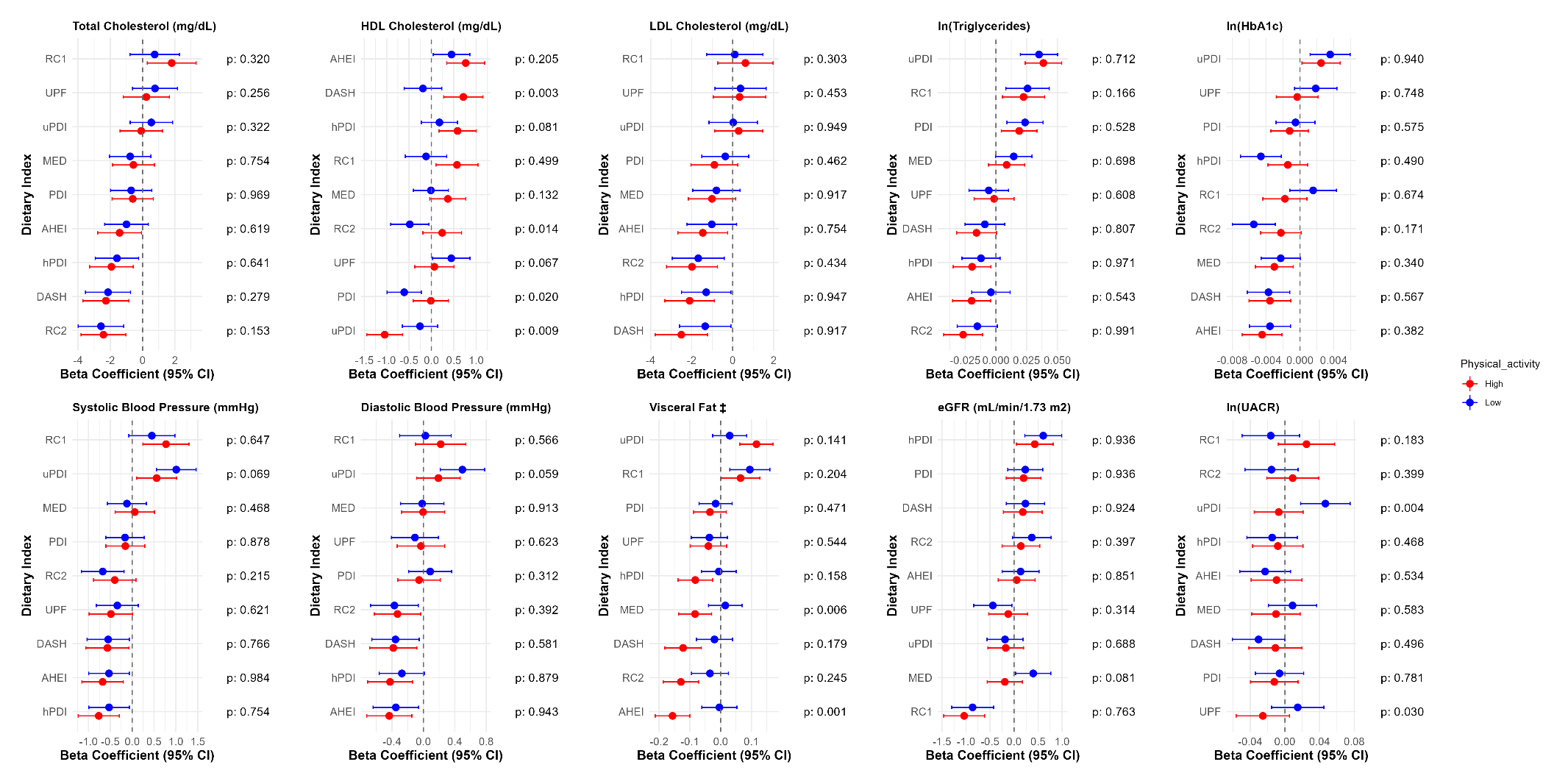


## Supplementary Figure S7. Subgroup analysis of the associations between dietary patterns and cardiovascular-kidney-metabolic risk markers by physical activity level (n_<median_=3738, n_>=median_=3717).

The forest plots show the beta-coefficients from the multivariable adjusted linear regression models for each of the dietary patterns analyzed continuously per 1 standard deviation increment (visually represented by the centers of the error bars), the 95% confidence intervals (visually represented by the error bars). The p‑value (p) shown originates from a separate interaction model and indicates the statistical significance of the interaction term. ‡ Visceral Fat Level index (range 1–30).


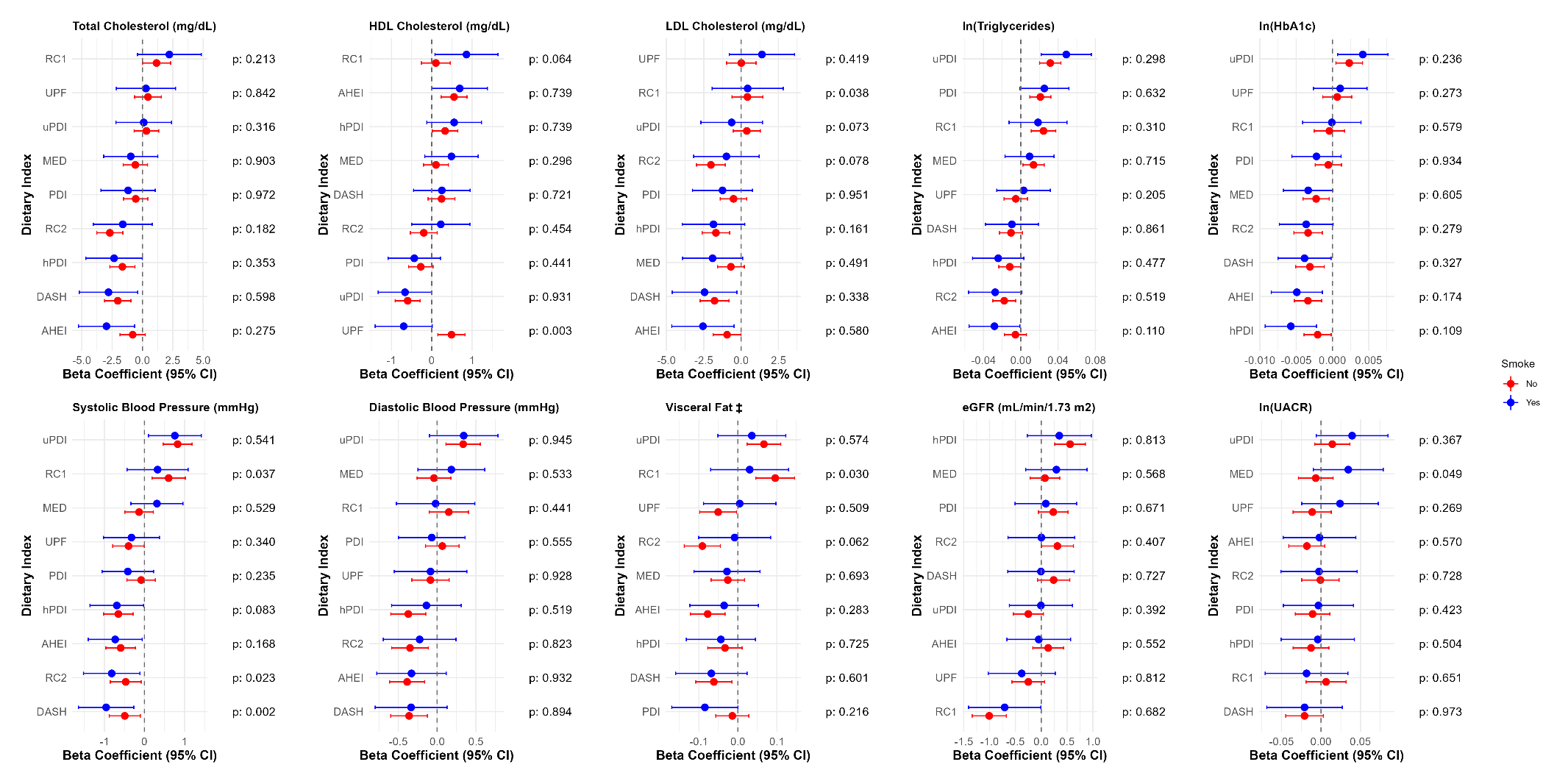


## Supplementary Figure S8. Subgroup analysis of the associations between dietary patterns and cardiovascular-kidney-metabolic risk markers by smoking status (n _never/former_=6617, n_current smokers_=1415).

The forest plots show the beta-coefficients from the multivariable adjusted linear regression models for each of the dietary patterns analyzed continuously per 1 standard deviation increment (visually represented by the centers of the error bars), the 95% confidence intervals (visually represented by the error bars The p‑value (p) shown originates from a separate interaction model and indicates the statistical significance of the interaction term. ‡ Visceral Fat Level index (range 1–30).
